# Synthesis, enantioseparation and photophysical properties of planar-chiral pillar[5]arene derivatives bearing fluorophore fragments

**DOI:** 10.3762/bjoc.15.164

**Published:** 2019-07-18

**Authors:** Guojuan Li, Chunying Fan, Guo Cheng, Wanhua Wu, Cheng Yang

**Affiliations:** 1 Key Laboratory of Green Chemistry & Technology, College of Chemistry and Healthy Food Evaluation Research Center, Sichuan University, 29 Wangjiang Road, Chengdu, 610064, P. R. China

**Keywords:** aggregation, circular dichroism, chirality, click chemistry, macrocycles, pillar[5]arenes

## Abstract

Planar chiral pillar[5]arene derivatives (**P5A-DPA** and **P5A-Py**) bearing bulky fluorophores were obtained in high yield by click reaction. The photophysical properties of both compounds were investigated in detail. **P5A-DPA** with two 9,10-diphenylanthracene (DPA) pigments grafted on the pillar[5]arene showed a high fluorescence quantum yield of 89.5%. This is comparable to the monomer **DPA-6**, while **P5A-Py** with two perylene (Py) pigments grafted on the pillar[5]arene showed a significantly reduced quantum yield of 46.4% vs 78.2% for the monomer **Py-6**. The oxygen-through-annulus rotation of the phenolic units was inhibited for both compounds due to the bulky chromophore introduced, and the resolution of the enantiomers was achieved due to the bulky size of the fluorophores. The absolute configuration of the enantiomers was determined by circular dichroism (CD) spectra. The solvent-induced aggregation behavior was investigated with the enantiopure **P5A-DPA** and **P5A-Py**. It was found that the CD signals were enhanced by aggregation. **P5A-DPA** showed aggregation-induced emission enhancement, while **P5A-Py** showed aggregation-induced emission quenching, accompanied by excimer emission when aggregating in water and THF mixed solution.

## Introduction

Planar-chiral compounds are structurally appealing and potentially applicable in various functional materials such as chiral discriminators [1–2], chiral polymers, supramolecular sensors [3] and chiral guest receptors [4–5]. Planar-chiral macrocyclic molecules are particularly interesting in the context of the host–guest complexation properties [6–8]. Pillararenes are typical examples of this type of compounds and have attracted considerable attention due to their facile chemical synthesis and versatile functionality [9] in recent years. Pillar[5]arenes [10], are cyclic pentamers composed of 1,4-dialkoxybenzene units connected through methylene bridges at the *para*-position and have a unique symmetrical architecture with an overall cylindrical or pillar-like shape. By virtue of their rigid and symmetrical structures, as well as highly tunable functionality, the studies on pillar[5]arenes have been developed rapidly in various areas, such as artificial transmembrane channels [11–12], molecular complexation [13–14] and nonporous adaptive crystals [15–17]. One of the features of pillar[5]arenes that differs from the common macrocycles is the planar chirality resulting from the different orientations of the alkoxy substituents on the rims. Theoretically, eight conformers can be formed including diastereomeric ones: (*S_p_,S_p_,S_p_,S_p_,S_p_*), (*R_p_,S_p_,S_p_,S_p_,S_p_*), (*R_p_,R_p_,S_p_,S_p_,S_p_*), (*R_p_,S_p_,R_p_,S_p_,S_p_*) and their antipodal enantiomers: (*R_p_,R_p_,R_p_,R_p_,R_p_*), (*S_p_,R_p_,R_p_,R_p_,R_p_*), (*S_p_,S_p_,R_p_,R_p_,R_p_*), (*S_p_,R_p_,S_p_,R_p_,R_p_*). Among them, the enantiomeric per-*S_p_* (*S_p_,S_p_,S_p_,S_p_,S_p_*) and per-*R_p_* (*R_p_,R_p_,R_p_,R_p_,R_p_*) conformers (abbreviated as *S_p_* and *R_p_*, respectively), in which all of the alkoxy substituents at both rims are oriented in the same direction, are the most stable conformers due to the steric-hindrance effect of the substituents in other conformers. In general, the *S_p_* and *R_p_* conformers can interconvert from each other in solution through the oxygen-through-annulus rotation along the methylene bridges [18], and the separation of these conformational isomers is impossible.

Inhibiting the rotation of the phenolic units is prerequisite to isolate the isomers. A complexation with a suitable guest molecule, such as a viologen derivative, can significantly slow down the rotation of the phenolic rings, and a conformational interconversion can only occur when the guest molecule decomplexed from the cavity [19–21]. Several strategies have been established to block the interconversion of the *S_p_* and *R_p_* conformers: Implanting a guest moiety in the pillar[5]arene cavity by rotaxanation or introducing a fused side ring into one of the phenolic rings [22–23]. Covalently connected bulky substituents installed on both rims of the pillar[n]arene provided a more straightforward method to inhibit the rotation [24–26]. In per-cyclohexylmethyl-substituted pillar[5]arene the rotation of the phenolic units is blocked and enabled the chiral separation of the *S_p_* and *R_p_* enantiomers [25]. Also installing bulky substituents onto only one phenolic ring was found to be effective to inhibit the rotation of the units [27–29].

**Scheme 1:**
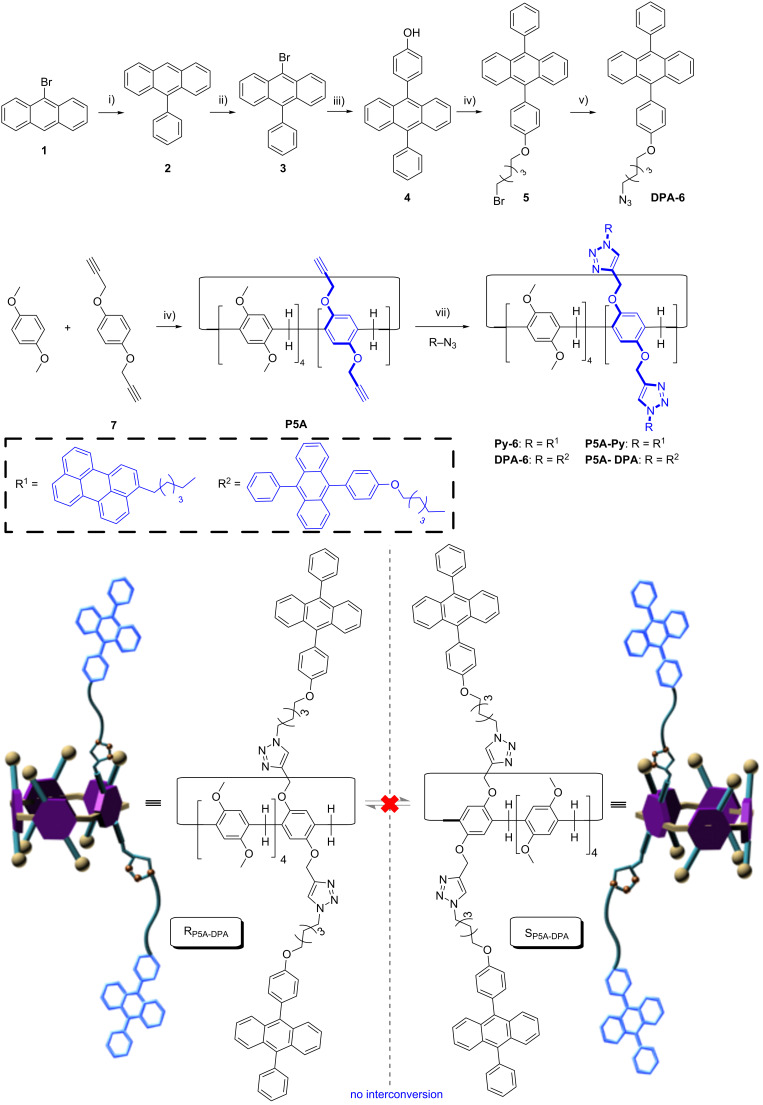
Preparation of A1/A2-difunctionalized pillar[5]arenes (**P5A-DPA** and **P5A-Py**) by click reactions. Reagents and conditions: i) phenylboronic acid, Pd(PPh_3_)_4_, K_2_CO_3_, CsF, toluene/THF/H_2_O, 120 °C, yield: 82%; ii) *N*-bromosuccinimide, chloroform, 60 °C, yield: 87%; iii) (4-hydroxyphenyl)boronic acid, Pd(PPh_3_)_4_, K_2_CO_3_, CsF, toluene/THF/H_2_O, reflux, 8 h, yield: 70%; iv) K_2_CO_3_, 1,5-dibromopentane, acetone, 80 °C, yield: 67%; v) NaN_3_, DMF, yield: 84%; vi) paraformaldehyde, BF_3_·OEt_2_, 1,2-dichloroethane, yield: 14%; vii) CuSO_4_·5H_2_O, sodium ascorbate, DMF, yield: 72%.

Pillar[5]arene itself shows only moderate absorption and weak fluorescence in the UV region, and the chemical introduction of chromophores onto the rims of pillar[5]arene has been applied to allow its use as receptors for molecular sensing or biological applications [30–33]. It occurred to us that connecting fluorophores with strong absorptions in the visible range and with a high fluorescence quantum yield are beneficial for the application of pillar[5]arene in these fields. Perylene (Py) and 9,10-diphenylanthracene (DPA) are well known for their desirable absorption and high fluorescence quantum yield. These chromophores possess unique photophysical properties and have been widely used as triplet acceptor for TTA-based upconversion [34–36] and as energy donors in artificial light-harvesting systems [37]. Thus, two pillar[5]arene derivatives **P5A-DPA** and **P5A-Py** (Scheme 1), in which two Py and DPA units were chemically grafted onto one of the phenolic units of pillar[5]arene, were designed and synthesized by click reaction [38–39]. We supposed that the Py or DPA substituents on both rims of the pillar[5]arenes will serve two purposes: Firstly, the molecular sizes of Py and DPA are larger than the cavity size of the pillar[5]arenes (ca. 5.5 Å), and the rotation of the units should be inhibited generating a pair of *S_p_* and *R_p_* enantiomers. Secondly, the strong absorption in the visible range and high fluorescence a quantum yield of the fluorophores will donate the pillar[5]arene derivatives novel photophysical properties.

## Results and Discussion

### Syntheses of pillar[5]arene derivatives

**P5A-Py** was synthesized by following our previously reported processes [34], and **P5A-DPA** was synthesized by the processes illustrated in Scheme 1. The ethynyl-bearing pillar[5]arene derivative **P5A** was obtained through the co-cyclization of 4.0 equiv of 1,4-dimethoxybenzene with 1.0 equiv of the hydroquinone derivative **7** and 5.0 equiv of paraformaldehyde in the presence of BF_3_·OEt_2_ [40–41]. The product was purified by silica gel column chromatography with hexane/ethyl acetate 10:1 as the eluent. The first fraction was permethylated pillar[5]arene, and **P5A** was collected as the second fraction in 45% yield. The subsequent reaction of compound **P5A** with the azide-substituted DPA derivative **DPA-6** was carried out in the presence of CuSO_4_·5H_2_O (2.0 equiv) and sodium ascorbate (4.0 equiv) for 24 h in DMF at 65 °C giving **P5A-DPA** in almost quantitative conversion (Scheme 1). The crude product was separated from CuSO_4_ and sodium ascorbate through extraction with Et_2_O/water. Further purification of the crude product was carried out by silica gel column chromatography (dichloromethane/methanol 30:1) to afford **P5A-DPA** in 72% yield. The structure of **P5A-DPA** was characterized by ^1^H NMR, ^13^C NMR and high-resolution mass spectrometry (HRMS, Supporting Information File 1, Figures S19–S21).

### Photophysical properties

**Figure 1:**
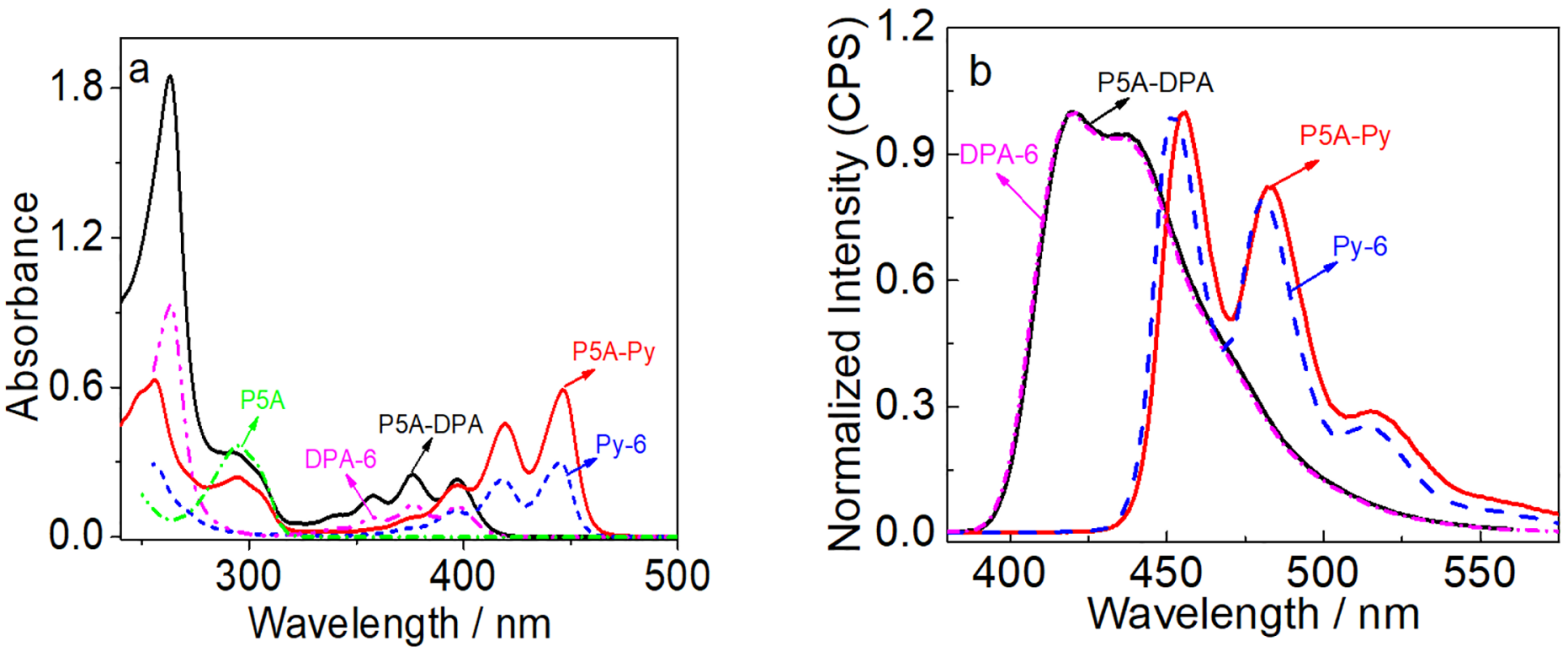
UV–vis absorption (a) and fluorescence emission spectra (b) of **Py-6**, **P5A-Py** (λ_ex_ = 420 nm) and **DPA-6**, **P5A-DPA** (λ_ex_ = 375 nm) in CHCl_3_, *c* = 1.0 × 10^−5^ M, 25 °C.

The photophysical properties were investigted by UV–vis absorption and fluorescence spectroscopy, as well as through fluorescence decay measurements. As shown in Figure 1a, the UV–vis spectrum of **P5A-DPA** showed two main absorption bands at 270–320 nm and 350–420 nm, respectively, which were assigned to the absorption bands of the pillar[5]arene moiety and the π→π* transition of the DPA fragment, respectively. The absorption spectra of **P5A-DPA** and **P5A-Py** are almost the result of the addition of the spectra of pillar[5]arene with **DPA-6** and **Py-6**. The molar extinction coefficient at the longer wavelength for **P5A-DPA** was almost double that of **DPA-6**, e.g., ε = 23100 M cm^−1^ for **P5A-DPA** vs 12200 M cm^−1^ for **DPA-6** at 397 nm, indicating that there is no strong interaction between the two DPA units in **P5A-DPA** at the ground state. Similar results were observed for **P5A-Py** except for a slight bathochromic shift of the absorption compared with **Py-6**.

The fluorescence spectrum of **P5A-DPA** is very similar to that of **DPA-6** (Figure 1b). **P5A-DPA** showed an intense fluorescence in the visible light region, peaked at 420 nm with a shoulder at 437 nm and a fluorescence quantum yield as high as 89.5%, which is very close to that of **DPA-6** (Φ_F_ = 92.1%). For **P5A-Py**, however, it showed a slightly red-shifted emission compared with **Py-6**, with the emission peaks at 456 nm and 483 nm and a shoulder at 516 nm (Table 1). The quantum yield was significantly decreased to 46.4% compared with 78.2% for **Py-6**. We ascribed the decreased fluorescence of **P5A-Py** to the π–π stacking of the Py units caused by the high local concentration of perylene. For **P5A-DPA**, which also bears two DPA units in one macrocyclic host, the fluorescent quantum yield was only slightly decreased. This should be mainly due to the steric hindrance of the 9- and 10-phenyl groups, which inhibited the π–π stacking of the anthracene core in DPA.

**Table 1:** Photophysical parameters of the synthesized monomers **Py-6**, **DPA-6** and the planar-chiral hosts **P5A-Py**, **P5A-DPA**.^a^

	λ_abs_ / nm	ε^b^	λ_em_ / nm	Φ_F_^c^	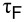 / ns^d^

**P5A-DPA**	295, 376, 397	3.04, 2.50, 2.31	420, 437	89.5	4.75
**DPA-6**	376, 397	1.30, 1.22	420, 435	92.1	4.95
**P5A-Py**	295, 419, 446	2.39, 4.53, 5.88	456, 483, 516	46.4^d^	3.36
**Py-6**	418, 445	2.31, 2.99	451, 480, 514	78.2^d^	4.40

^a^In CHCl_3_ at 1.0 × 10^−5^ M. ^b^Molar extinction coefficient at the absorption maxima (10^4^ M^−1^ cm^−1^). ^c^Fluorescence quantum yields estimated by a relative method using DPA (Φ_F_ = 95% in ethanol) as the standard. ^d^Perylene (Φ_F_ = 98% in *n*-hexane) as the standard. ^d^Fluorescence lifetimes.

**Figure 2:**
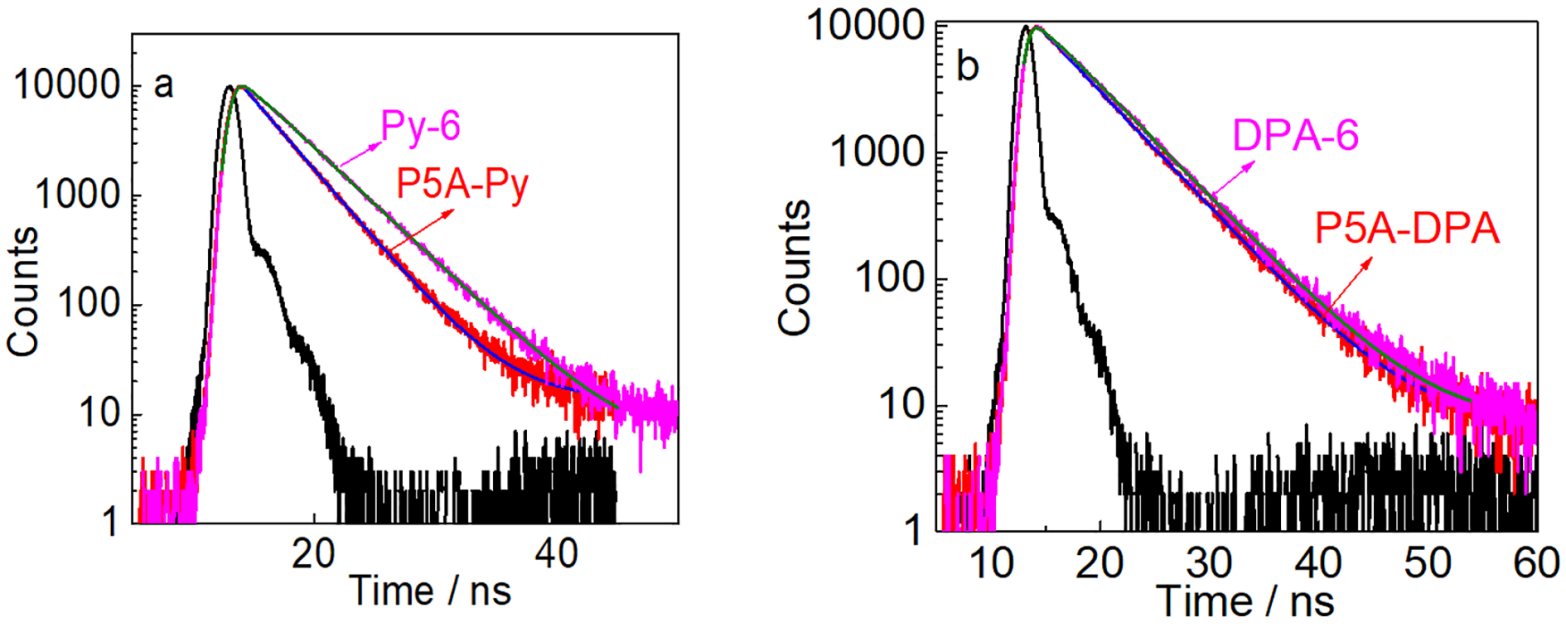
(a) Fluorescence decay curves of **Py-6 and P5A-Py** at 450 nm and (b) fluorescence decay curves of **DPA-6** and **P5A-DPA** at 420 nm, excited at 390 nm nanosecond LED, *c* = 1.0 × 10^−5^ M in CHCl_3_, 25 °C.

The fluorescent lifetimes of **P5A-Py** and **P5A-DPA** were compared with **Py-6** and **DPA-6**. As shown in Figure 2, the lifetime of **P5A-Py** is 3.4 ns which is shorter than that of **Py-6** (4.4 ns), demonstrating that grafting two Py units in close proximity in one host, opened a new route for nonradiative decay. This phenomenon further verified the occurrence of π–π stacking of the Py fragments in **P5A-Py**. The lifetime of **P5A-DPA** was 4.8 ns, which is very similar to **DPA-6** (5.0 ns) and the non-substituted DPA (5.3 ns) [42]. The fact that grafting DPA units in one host did not influence the fluorescent quantum yield together with the appealing host–guest properties make **P5A-DPA** an ideal candidate for applications as acceptor for triplet–triplet annihilation upconversion.

### Optical properties

**Figure 3:**
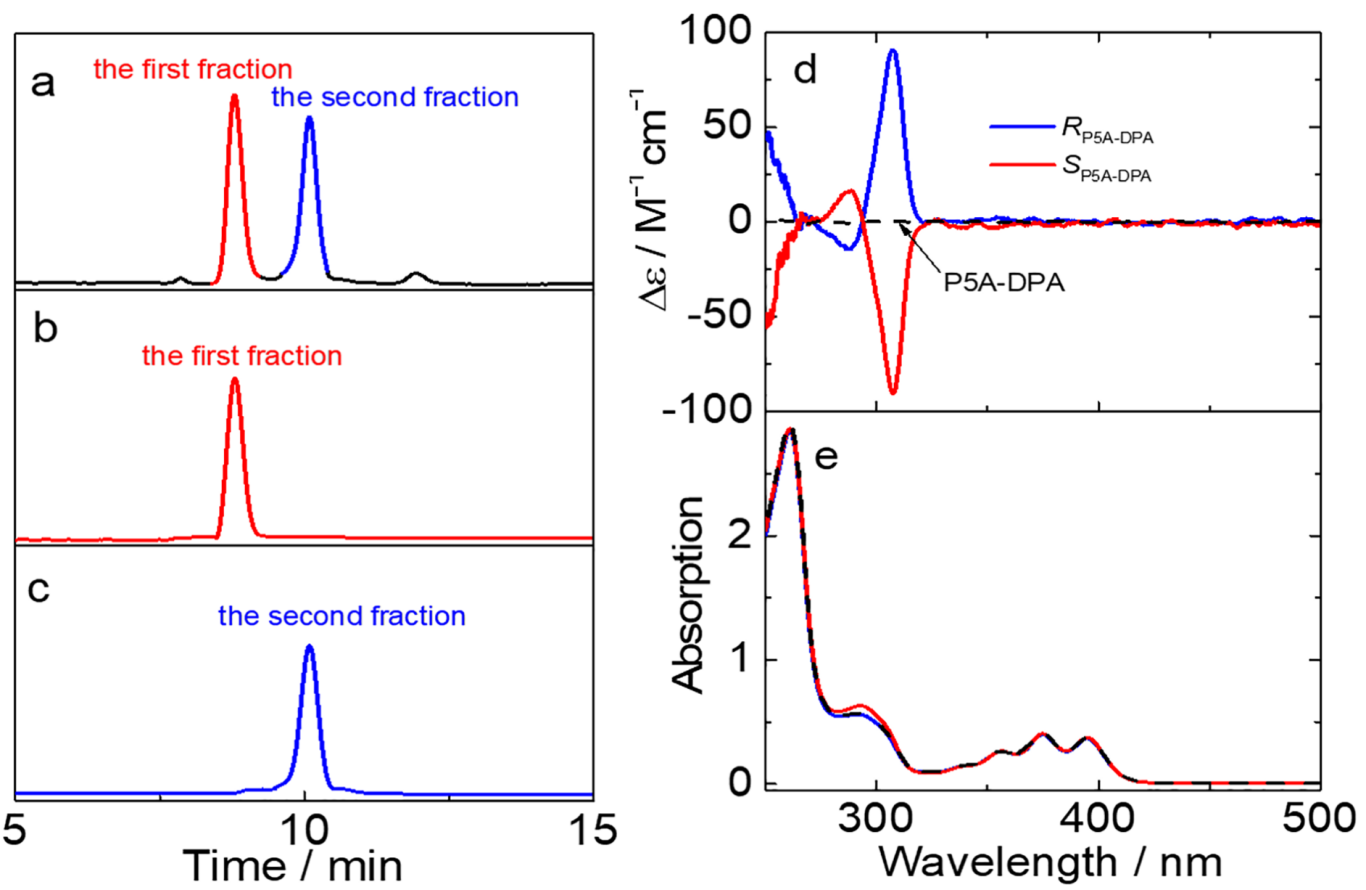
(a) Chiral HPLC traces of **P5A-DPA**, (b), (c) the first and second fractions of **P5A-DPA**, detected by UV at 295 nm. Conditions: column, DAICEL CHIRALPAK IA; mobile phase: hexane/dichloromethane 35:65; flow rate = 4.0 mL/min; temperature 25 °C). (d) CD and (e) UV–vis spectra of racemic **P5A-DPA**, the first and second fractions (10 μM) in CHCl_3_ at 25 °C.

Due to the fact that the Py and DPA units are too large to enter the cavity of the pillar[5]arene, we expected that the oxygen-through-annulus rotation is blocked in **P5A-DPA** and **P5A-Py**, giving rise to two pairs of enantiomeric pillar[5]arene derivatives. The racemic mixtures of **P5A-DPA** and **P5A-Py** were respectively separated by chiral high-performance liquid chromatography (HPLC). As shown in Figure 3a, injection of a **P5A-DPA** solution onto a DAICEL CHIRALPAK IA chiral HPLC column afforded two well-separated peaks of almost equal areas at 8.8 min and 10.0 min, respectively, demonstrating that **P5A-DPA** was the racemic mixture of *R_p_* and *S_p_* configuration. The two fractions were collected separately and re-injected into the chiral column to confirm the enantiomeric purity and to check if racemization of the enantiomers takes place in solution at room temperature. In both cases, only the original peak was detected and the peak for the antipodal enantiomer was not detected, indicating that no racemization of the enantiomer of **P5A-DPA** takes place at room temperature (Figure 3b and c). **P5A-Py** showed a similar phenomenon (Figure S22a,b and c). These results demonstrated that the bulky substituents DPA or perylene effectively prevent the ring rotations, and therefore making the separation of *R_p_* and *S_p_* conformers possible.

Figure 3d and e show the circular dichroism (CD) and UV–vis spectra of each fraction for **P5A-DPA**. The CD signals of both fractions observed at 270–320 nm are perfect mirror images, thus confirming that the two fractions contain a pair of enantiomers. The positive Cotton effect observed at ca. 310 nm was assigned to the *R_p_* configured enantiomer whereas the negative Cotton effect at ca. 310 nm was assigned to the corresponding *S_p_* configured isomer, based on calculations as well as single-crystal X-ray diffraction published recently [20]. Thus, the absolute configuration of the first fraction was assigned to be *S_p_* and the second fraction was assigned to the *R_p_* isomer. Unexpectedly, no induced CD signals of DPA units at 360–420 nm were observed though **P5A-DPA** showed a strong absorption (with ε > 10^4^ M^−1^ cm^−1^, Figure 3e) in this region. The same is true for **P5A-Py** (Supporting Information File 1, Figure S22d and e). We attributed the absence of induced CD signals to the long distances of the fluorophores to the chiral center and therefore chirality transfer is non-effective [43].

**Figure 4:**
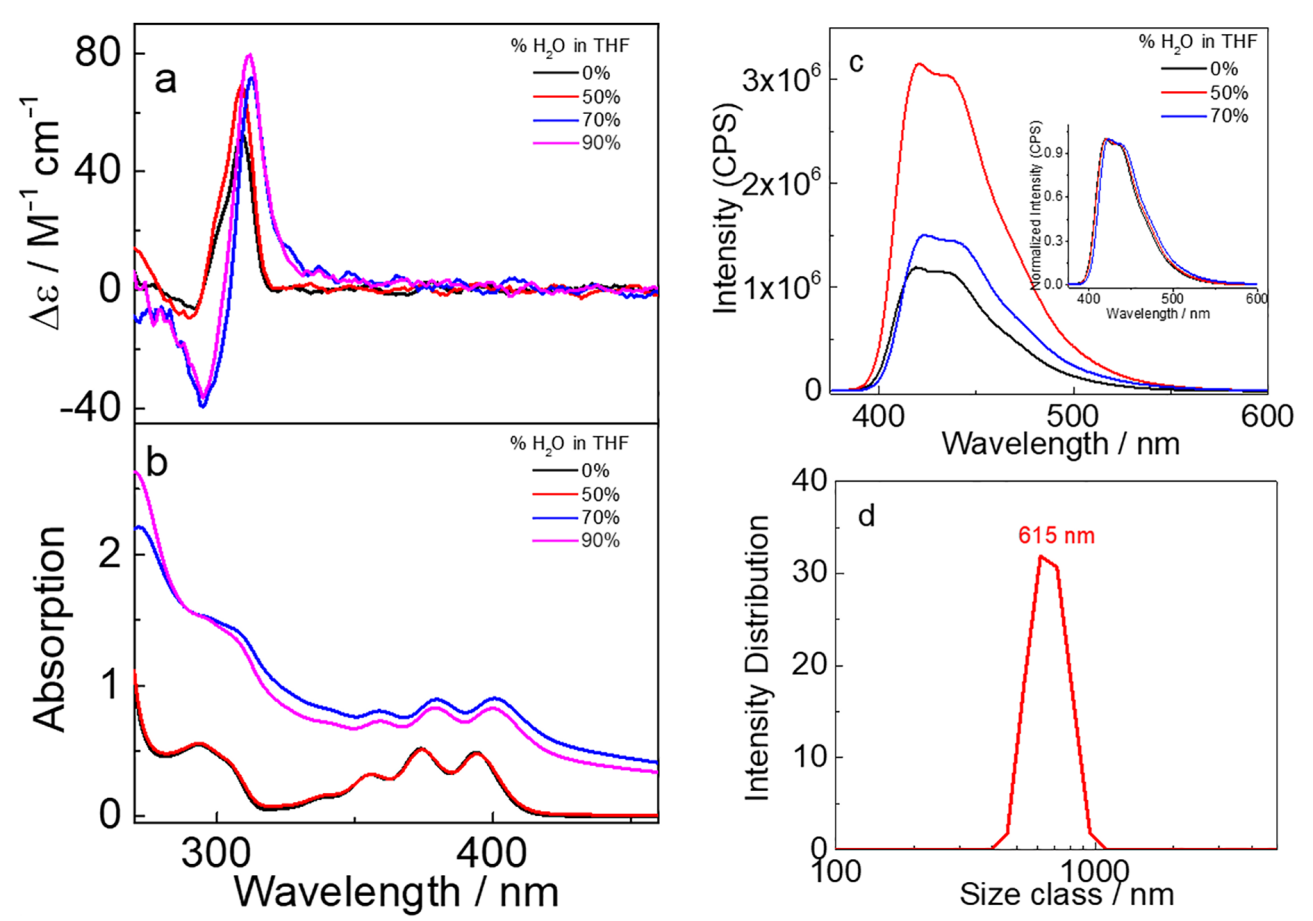
(a) CD, (b) UV–vis and (c) fluorescence spectra of the *R***_P5A-DPA_** (20 μM) in THF and THF/H_2_O solvent mixtures; inset: the normalized fluorescence spectrum. (d) DLS measurements of the aggregated *R***_P5A-DPA_** obtained from the mixture of THF/H_2_O 1:1 (v/v).

Supramolecular assembly usually leads to different photophysical properties than homogeneous solutions. We have demonstrated that solvents play a critical role in chiral recognition and chiral photoreactions [44–51]. We therefore further investigated the solvent-induced aggregation behaviors of the chirally pure conformers by adding different proportions of water into their THF solutions. Taking the *R_p_* conformers for example, the aggregation of **P5A-DPA** is apparently divided into two stages (Figure 4). By adding 50% water to THF, the UV–vis absorption wavelength of **P5A-DPA** did not change but the CD signals were obviously enhanced. This is most probably due to the formation of aggregates and the rotation of the phenolic units in pillar[5]arene host was inhibited in the aggregates. The aggregation behavior was further investigated by dynamic light scattering (DLS) analyses, and revealed the formation of nanoparticles with 615 nm in average diameter after the addition of 50% water (Figure 4d). Increasing the amount of water to 70% or 90% led to a bathochromic shift of the UV–vis absorption and a raise of the baseline, indicating particles of larger size were formed. The CD signals continuously increased accompanied with a red-shifting of the wavelength, thus demonstrating that the DPA pigments started to aggregate in solutions with a water content of more than 50%. The π–π stacking of the anthracene core in DPA is responsible for the red-shift of the absorption. For **P5A-Py**, however, adding water to the THF solution led to a continuous red-shifting of the UV–vis spectra and increasing of CD signal, which is reasonable, as the planar structure of Py tends more to get aggregated in water by π–π stacking. Aggregates with an average diameter of 531 nm are formed in 60% water (Figure S23, Supporting Information File 1) [52]. Exciton coupling CD (ECCD) signals were not observed for both compounds, even when adding more than 90% water, demonstrating that the chromophores DPA or Py units were not asymmetrically oriented in the aggregates.

Interestingly, by adding water into the THF solution of **P5A-DPA**, the fluorescence intensity firstly increased by 3 times at 50% water and then decreased when the water content was increased to 70% (Figure 4c). We attributed the enhanced emission of **P5A-DPA** in the presence of less than 50% water to an inhibited rotation of the 9,10-phenyl groups in the DPA units, which reduced the inactivation of the excited state through non-radiative transition [53]. However, when increasing the amount of water, the fluorescence starts to decrease due to the π–π stacking of DPA, which led to aggregation-caused quenching (ACQ), as was demonstrated by UV–vis absorption and CD studies. For compound **P5A-Py**, however, the fluorescence intensity continuously decreased when adding water into the THF solutions, and at 70% water content, a new emission peak at 540 nm was observed which was assigned to the excimer emission of Py (Figure S23, Supporting Information File 1) [54].

## Conclusion

Two new planar chiral macrocyclic hosts **P5A-DPA** and **P5A-Py** were synthesized by grafting two fluorophore pigments (DPA or Py) on pillar[5]arene through CuAAC “click” reaction. The new macrocyclic compounds showed strong absorptions in the visible range and high fluorescence quantum yield, e.g., **P5A-DPA** showed absorption at 350–410 nm and a quantum yield of 89.5% which is comparable to the monomer **DPA-6**. On the other hand, **P5A-Py** with two perylene (Py) pigments grafted on the pillar[5]arene showed intense absorption at 385–460 nm and a quantum yield of 46.4%. The rotation of phenolic units was inhibited by introducing two bulky fluorophores in one of the phenolic units of pillar[5]arene, and the enantiomers were isolated by chiral HPLC. The absolute configurations of each fraction were determined by circular dichroism (CD) spectroscopy. The first fraction showing negative CD signals was assigned to the *S_p_* conformer and the second fraction showing positive CD signals was assigned to the *R_p_* conformer. Aggregation behaviors of the enantiopure chiral pillar[5]arene derivatives were investigated by adding water into the THF solution. **P5A-DPA** and **P5A-Py** showed an enhancement of the CD signals by adding water due to restriction of the conformers’ rotations by aggregation. **P5A-DPA** showed a fluorescence enhancement when less than 50% of water was added, while **P5A-Py** showed emission quenching and excimer emission when adding water into the THF solution. This work presented a new strategy for achieving versatile planar chiral hosts, and these hosts will have potential applications in various fields such as supramolecular sensing, host–guest recognition and triplet–triplet annihilation upconversion.

## Experimental

### General materials and methods

All reagents and chemicals used in synthesis were analytically pure and used as received without further purification. ^1^H NMR and ^13^C NMR spectra were recorded at room temperature on a Bruker AMX–400 spectrometer (operating at 400 MHz for ^1^H NMR and 100 MHz for ^13^C NMR) in CDCl_3_ with TMS as an internal standard. Due to the poor solubility, NMR spectra of **P5A-DPA** were recorded at room temperature on a Bruker AMX-600 spectrometer (operating at 600 MHz for ^1^H NMR and 151 MHz ^13^C NMR) in CDCl_3_. High-resolution mass spectra (HRMS) were measured using a Waters-Q-TOF Premiers (ESI) apparatus. UV–vis spectra were obtained on a JASCO v-650 spectrometer. Fluorescence spectra and fluorescence lifetime decay measurements were recorded on a HORIBA FluoroMax–4 (TCSPC) spectrofluorimeter. Circular dichroism spectra were measured on a JASCO J-1500 spectrometer using a quartz cuvette of 1 cm path length installed in a Unisoku cryostat. Dynamic light scattering (DLS) studies were done on a Zetasizer Nano ZS90 instrument. A preparative Chiralpak IA column was used for the separation of enantiomers.

### Synthesis and characterization of **P5A-DPA**

The synthesis and characterization data of **P5A-Py** have been reported elsewhere [34].

**Synthesis of 2:** In a similar manner as described previously [55]. An aqueous solution of K_2_CO_3_ (5.4 g, 39.0 mmol) and CsF (3.7 g, 24.3 mmol) was added to the solution of compound **1** (5.0 g, 19.5 mmol) and phenylboronic acid (3.6 g, 25.4 mmol) in the mixed solvent of toluene (40 mL), tetrahydrofuran (60 mL) and water (10 mL). After bubbling argon through the mixture for 15 min, Pd(PPh_3_)_4_ (300 mg, 0.3 mmol) was added, and the mixture was stirred and refluxed for 8 h. Then, the reaction mixture was extracted with dichloromethane, and the organic layer was dried over anhydrous Na_2_SO_4_. The solution was concentrated under reduced pressure to obtain the crude product which was further purified by chromatography (silica gel, dichloromethane/petroleum ether 30:1) to afford compound **2** as white solid (4.8 g, 82.0%). Mp 156 °C; the ^1^H NMR spectrum of **2** is shown in Figure S1, Supporting Information File 1. ^1^H NMR (400 MHz, CDCl_3_) 8.49 (s, 1H), 8.04 (d, *J* = 8.5 Hz, 2H), 7.67 (d, *J* = 8.8 Hz, 2H), 7.60–7.51 (m, 3H), 7.47–7.42 (m, 4H), 7.36–7.34 (m, 2H). The ^13^C NMR spectrum of **2** is shown in Figure S2, Supporting Information File 1. ^13^C NMR (100 MHz, CDCl_3_) 138.76, 137.01, 130.33, 131.22, 130.18, 128.34, 128.30, 127.42, 126.81, 126.52, 125.30, 125.07. The electrospray ionization mass spectrum of **2** is shown in Figure S3, Supporting Information File 1. HRESIMS (*m*/*z*): [M]^+^ calcd for [C_20_H_14_]^+^, 254.1096, found, 254.1125; [M + Na]^+^ calcd for [C_20_H_14_Na]^+^, 277.0993; found, 277.1268.

**Synthesis of 3:** In a Schlenk tube under argon, compound **2** (2.5 g, 10.0 mmol) and *N*-bromobutanimide (2.1 g, 12.0 mmol) were dissolved in chloroform (30.0 mL). The mixture was heated at 60 °C for 2 h and then the solvent evaporated under reduced pressure. Afterwards, dichloromethane and water were added, and the organic layer was collected and dried over anhydrous Na_2_SO_4_. The crude product was purified by column chromatography using dichloromethane/petroleum ether 20:1 as the eluent to yield **3** as white solid (2.8 g, 87%). Mp 154–155 °C. The ^1^H NMR spectrum of **3** is shown in Figure S4, Supporting Information File 1. ^1^H NMR (400 MHz, CDCl_3_) 8.61 (d, *J* = 8.9 Hz, 2H), 7.64 (d, *J* = 8.7 Hz, 2H), 7.61–7.53 (m, 5H), 7.42–7.33 (m, 4H). The ^13^C NMR spectrum of **3** is shown in Figure S5, Supporting Information File 1. ^13^C NMR (100 MHz, CDCl_3_) 138.38, 137.79, 131.12, 131.02, 130.22, 128.43, 127.83, 127.73, 127.47, 127.38, 126.93, 125.52, 122.71. The electrospray ionization mass spectrum of **3** is shown in Figure S6, Supporting Information File 1. HRESIMS (*m*/*z*): [M]^+^ calcd for [C_20_H_13_Br]^+^, 332.0201; found, 332.1679.

**Synthesis of 4:** Compound **4** was prepared by a similar procedure as that used for **2**, but with **3** (1.5 g, 70%) as the starting material. The ^1^H NMR spectrum of **4** is shown in Figure S7, Supporting Information File 1. Mp 245–247 °C; ^1^H NMR (400 MHz, CDCl_3_) 7.77–7.72 (m, 2H), 7.71–7.65 (m, 2H), 7.63–7.57 (m, 2H), 7.57–7.52 (m, 1H), 7.48–7.46 (m, 2H), 7.37–7.30 (m, 6H), 7.09–7.04 (m, 2H). The ^13^C NMR spectrum of **4** is shown in Figure S8, Supporting Information File 1. ^13^C NMR (100 MHz, CDCl_3_) 154.97, 139.08, 136.98, 136.75, 132.57, 131.31, 130.18, 129.89, 129.66, 128.39, 127.44, 126.97, 126.94, 124.97, 124.94, 120.78, 115.35, 115.27. The electrospray ionization mass spectrum of **4** is shown in Figure S9, Supporting Information File 1. HRESIMS (*m*/*z*): [M − H]^−^ calcd for [C_26_H_17_O]^−^, 345.1279; found, 345.1277.

**Synthesis of 5:** Under nitrogen atmosphere, a mixture of compound **4** (800.0 mg, 2.3 mmol), K_2_CO_3_ (952.2 mg, 6.9 mmol) and 1,5-dibromopentane (1.1 g, 4.6 mmol) was dissolved in 30 mL dry acetone and the mixture was heated under reflux for 30 h. After cooling, the solvents were removed under reduced pressure and the residue was purified by column chromatography on silica gel (100–200 mesh) using a mixture dichloromethane/petroleum ether 1:1 as the eluent to give compound **5** in 67% yield, 760.8 mg. Mp 155.6–161.1 °C. The ^1^H NMR spectrum of **5** is shown in Figure S10, Supporting Information File 1. ^1^H NMR (400 MHz, CDCl_3_) 7.78–7.72 (m, 2H), 7.71–7.66 (m, 2H), 7.58 (d, *J* = 15.4 Hz, 3H), 7.49–7.45 (m, 2H), 7.34 (d, *J* = 13.6 Hz, 6H), 7.12 (d, *J* = 8.7 Hz, 2H), 4.12 (t, *J* = 6.3 Hz, 2H), 3.50 (t, *J* = 6.8 Hz, 2H), 2.02 (d, *J* = 14.7 Hz, 2H), 1.96–1.88 (m, 2H), 1.73 (d, *J* = 8.5 Hz, 2H). The ^13^C NMR spectrum of **5** is shown in Figure S11, Supporting Information File 1. ^13^C NMR (100 MHz, CDCl_3_) 158.46, 139.15, 136.97, 136.95, 132.41, 131.36, 131.07, 130.23, 129.93, 128.43, 127.47, 127.08, 126.98, 124.99, 124.93, 114.40, 67.70, 33.71, 32.59, 28.62, 25.01. The electrospray ionization mass spectrum of **4** is shown in Figure S12, Supporting Information File 1. HRESIMS (*m*/*z*): [M]^+^ calcd for [C_31_H_27_BrO]^+^, 494.1245; found, 494.1265; [M + H]^+^ calcd for [C_31_H_28_BrO]^+^, 495.1324; found, 495.1264.

**Synthesis of DPA-6:** To a solution of compound **5** (400.0 mg, 0.81 mmol) in DMF (15 mL) was added NaN_3_ (510.3 mg, 8.1 mmol). The mixture was stirred at 45 °C for 4 h (monitored by TLC until complete consumption of the starting material). The reaction mixture was poured into water and the aqueous layer was extracted with ether (3 × 10 mL). The combined organic layers were dried over Na_2_SO_4_ and concentrated in vacuo. The residue was purified by column chromatography on silica-gel (eluent: dichloromethane/petroleum ether 1:1) to give compound **DPA-6** as pale yellow solid (310.8 mg, 84%). Mp 140.2 °C. The ^1^H NMR spectrum of **DPA-6** is shown in Figure S13, Supporting Information File 1. ^1^H NMR (400 MHz, CDCl_3_) 7.81–7.74 (m, 2H), 7.72 (d, *J* = 7.4 Hz, 2H), 7.65–7.53 (m, 3H), 7.53–7.46 (m, 2H), 7.44–7.31 (m, 6H), 7.17–7.11 (m, 2H), 4.14 (t, *J* = 6.3 Hz, 2H), 3.39 (t, *J* = 6.7 Hz, 2H), 2.00–1.89 (m, 2H), 1.78 (d, *J* = 13.3 Hz, 2H), 1.72–1.63 (m, 2H). The ^13^C NMR spectrum of **DPA-6** is shown in Figure S14, Supporting Information File 1. ^13^C NMR (100 MHz, CDCl_3_) 158.48, 139.16, 136.97, 132.43, 131.37, 131.07, 130.25, 129.95, 128.45, 127.48, 127.10, 127.05, 125.01, 124.95, 114.40, 67.69, 51.46, 29.01, 28.78, 23.57. The electrospray ionization mass spectrum of **DPA-6** is shown in Figure S15, Supporting Information File 1. HRESIMS (*m*/*z*): [M]^+^ calcd for [C_31_H_27_N_3_O]^+^), 457.2154; found, 457.2150; [M + H]^+^ calcd for [C_31_H_28_N_3_O]^+^, 458.2232; found, 458.2213.

**Synthesis of P5A:** Analogous as previously described [56]. To a solution of 1, 4-dimethoxybenzene (1.7 g, 1.2 mmol) and **7** (55.8 mg, 0.3 mmol) in 1,2-dichloroethane (150 mL), paraformaldehyde (271.5 mg, 3.0 mmol) was added under nitrogen atmosphere. Then, boron trifluoride diethyl etherate (120 μL) was added to the solution and the mixture was stirred at room temperature for 1 h. Water (100 mL) was added to quench the reaction. The mixture was filtered and the solvent was removed. The residue was dissolved in dichloromethane. The organic layer was dried over anhydrous Na_2_SO_4_ and the solvent was evaporated to afford the crude product, which was isolated by flash column chromatography using ethyl acetate/petroleum ether 1:5 (*v*/*v*) as the eluent to give **P5A** as white solid (108.8 mg, 14%). The ^1^H NMR spectrum of **P5A** is shown in Figure S16, Supporting Information File 1. ^1^H NMR (400 MHz, CDCl_3_) 6.82 (s, 2H), 6.80 (s, 2H), 6.79 (s, 2H), 6.76 (s, 2H), 6.73 (s, 2H), 4.49 (d, *J* = 2.3 Hz, 4H), 3.80–3.76 (m, 10H), 3.70–3.64 (m, 24H), 2.02 (t, *J* = 2.3 Hz, 2H). The ^13^C NMR spectrum of **P5A** is shown in Figure S17, Supporting Information File 1. ^13^C NMR (100 MHz, CDCl_3_) 150.71, 150.67, 150.64, 150.57, 149.33, 129.09, 128.37, 128.31, 128.03, 127.85, 115.74, 114.23, 113.96, 113.86, 113.71, 78.87, 74.72, 56.36, 55.83, 55.79, 55.64, 29.69, 29.61. The electrospray ionization mass spectrum of **P5A** is shown in Figure S18, Supporting Information File 1. HRESIMS (*m*/*z*): [M + Na]^+^ calcd for [C_49_H_50_NaO_10_]^+^, 821.3302; found, 821.3292.

**Synthesis of P5A-DPA:** A mixture of compound **P5A** (20.0 mg, 0.02 mmol), **DPA-6** (47.2 mg, 0.1 mmol), CuSO_4_·5H_2_O (12.0 mg, 0.05 mmol), and sodium ascorbate (19.0 mg, 0.1 mmol) in DMF (5 mL) was refluxed under N_2_ atmosphere for 48 hours. The solvent was removed by rotary evaporation and the residue was purified by chromatography on silica gel (dichloromethane/ethyl acetate 30:1) to yield a brown solid (30.0 mg, 72%). The ^1^H NMR spectrum of **P5A-DPA** is shown in Figure S19, Supporting Information File 1. ^1^H NMR (600 MHz, CDCl_3_) 7.71 (m, *J* = 10.6 Hz, 8H), 7.57 (m, *J* = 7.2 Hz, 6H), 7.51–7.44 (m, 4H), 7.34 (m, *J* = 9.9 Hz, 12H), 7.22–6.75 (m, 14H), 4.07–3.39 (m, 40H), 2.97 (m, 4H), 2.14–1.59 (m, 14H). The ^13^C NMR spectrum of **P5A-DPA** is shown in Figure S20, Supporting Information File 1. ^13^C NMR (151 MHz, CDCl_3_) 171.17, 150.96, 139.08, 137.03, 136.89, 132.39, 131.33, 130.22, 129.96, 128.45, 127.50, 127.06, 126.92, 125.01, 124.98, 114.33, 113.82, 99.99, 67.49, 60.42, 58.48, 56.23, 55.72, 53.41, 31.95, 31.46, 30.21, 29.72, 29.68, 29.41, 29.38, 29.01, 29.00, 22.71, 21.07, 18.44, 14.22. The electrospray ionization mass spectrum of **P5A-DPA** is shown in Figure S21, Supporting Information File 1. HRESIMS (*m*/*z*): [M + Na]^+^ calcd for [C_111_H_104_N_6_NaO_12_]^+^, 1736.7643; found, 1736.7744; [M + K]^+^ calcd for [C_111_H_104_N_6_KO_12_]^+^, 1753.1813; found, 1753.7510.

## Supporting Information

File 1 Characterization spectra of all compounds, chiral HPLC traces of **P5A-Py**, CD and UV–vis spectra of the two fractions **P5A-Py** and the aggregation behaviors of **P5A-Py**.

## References

[1] Ôi S, Miyano S (1992). Chem Lett.

[2] Hattori T, Harada N, Oi S, Abe H, Miyano S (1995). Tetrahedron: Asymmetry.

[3] Fiesel R, Huber J, Scherf U (1996). Angew Chem, Int Ed Engl.

[4] Fiesel R, Huber J, Apel U, Enkelmann V, Hentschke R, Scherf U, Cabrera K (1997). Macromol Chem Phys.

[5] Katoono R, Kawai H, Fujiwara K, Suzuki T (2004). Tetrahedron Lett.

[6] Wei X, Wu W, Matsushita R, Yan Z, Zhou D, Chruma J J, Nishijima M, Fukuhara G, Mori T, Inoue Y (2018). J Am Chem Soc.

[7] Rao M, Kanagaraj K, Fan C, Ji J, Xiao C, Wei X, Wu W, Yang C (2018). Org Lett.

[8] Rao M, Wu W, Yang C (2019). Molecules.

[9] Ogoshi T, Yamafuji D, Kotera D, Aoki T, Fujinami S, Yamagishi T-a (2012). J Org Chem.

[10] Ogoshi T, Kanai S, Fujinami S, Yamagishi T-a, Nakamoto Y (2008). J Am Chem Soc.

[11] Si W, Chen L, Hu X-B, Tang G, Chen Z, Hou J-L, Li Z-T (2011). Angew Chem, Int Ed.

[12] Hu X-B, Chen Z, Tang G, Hou J-L, Li Z-T (2012). J Am Chem Soc.

[13] Li H, Chen D-X, Sun Y-L, Zheng Y B, Tan L-L, Weiss P S, Yang Y-W (2013). J Am Chem Soc.

[14] Wei P, Li D, Shi B, Wang Q, Huang F (2015). Chem Commun.

[15] Jie K, Zhou Y, Li E, Huang F (2018). Acc Chem Res.

[16] Jie K, Zhou Y, Li E, Zhao R, Huang F (2018). Angew Chem, Int Ed.

[17] Li E, Zhou Y, Zhao R, Jie K, Huang F (2019). Angew Chem, Int Ed.

[18] Ogoshi T, Kitajima K, Aoki T, Yamagishi T-a, Nakamoto Y (2010). J Phys Chem Lett.

[19] Gui J-C, Yan Z-Q, Peng Y, Yi J-G, Zhou D-Y, Su D, Zhong Z-H, Gao G-W, Wu W-H, Yang C (2016). Chin Chem Lett.

[20] Yao J, Wu W, Liang W, Feng Y, Zhou D, Chruma J J, Fukuhara G, Mori T, Inoue Y, Yang C (2017). Angew Chem, Int Ed.

[21] Lv Y, Xiao C, Yang C (2018). New J Chem.

[22] Ogoshi T, Yamafuji D, Aoki T, Kitajima K, Yamagishi T-a, Hayashi Y, Kawauchi S (2012). Chem – Eur J.

[23] Kitajima K, Ogoshi T, Yamagishi T-a (2014). Chem Commun.

[24] Ogoshi T, Kitajima K, Aoki T, Fujinami S, Yamagishi T-a, Nakamoto Y (2010). J Org Chem.

[25] Ogoshi T, Masaki K, Shiga R, Kitajima K, Yamagishi T-a (2011). Org Lett.

[26] Nierengarten I, Buffet K, Holler M, Vincent S P, Nierengarten J-F (2013). Tetrahedron Lett.

[27] Ogoshi T, Yamafuji D, Akutsu T, Naito M, Yamagishi T-a (2013). Chem Commun.

[28] Strutt N L, Schneebeli S T, Stoddart J F (2013). Supramol Chem.

[29] Strutt N L, Fairen-Jimenez D, Iehl J, Lalonde M B, Snurr R Q, Farha O K, Hupp J T, Stoddart J F (2012). J Am Chem Soc.

[30] Mastai Y (2009). Chem Soc Rev.

[31] Wu X, Gao L, Hu X-Y, Wang L (2016). Chem Rec.

[32] Feng W, Jin M, Yang K, Pei Y, Pei Z (2018). Chem Commun.

[33] Hu X-Y, Gao L, Mosel S, Ehlers M, Zellermann E, Jiang H, Knauer S K, Wang L, Schmuck C (2018). Small.

[34] Fan C, Wu W, Chruma J J, Zhao J, Yang C (2016). J Am Chem Soc.

[35] Xu W, Liang W, Wu W, Fan C, Rao M, Su D, Zhong Z, Yang C (2018). Chem – Eur J.

[36] Ogawa T, Yanai N, Monguzzi A, Kimizuka N (2015). Sci Rep.

[37] Peng H-Q, Chen Y-Z, Zhao Y, Yang Q-Z, Wu L-Z, Tung C-H, Zhang L-P, Tong Q-X (2012). Angew Chem, Int Ed.

[38] Yu G, Zhang Z, Han C, Xue M, Zhou Q, Huang F (2012). Chem Commun.

[39] Nierengarten I, Guerra S, Holler M, Nierengarten J F, Deschenaux R (2012). Chem Commun.

[40] Cao D, Kou Y, Liang J, Chen Z, Wang L, Meier H (2009). Angew Chem, Int Ed.

[41] Liu L, Cao D, Jin Y, Tao H, Kou Y, Meier H (2011). Org Biomol Chem.

[42] Xu K, Zhao J, Escudero D, Mahmood Z, Jacquemin D (2015). J Phys Chem C.

[43] Ogoshi T, Yamafuji D, Yamagishi T-a, Brouwer A M (2013). Chem Commun.

[44] Yan Z, Huang Q, Liang W, Yu X, Zhou D, Wu W, Chruma J J, Yang C (2017). Org Lett.

[45] Dai L, Wu W, Liang W, Chen W, Yu X, Ji J, Xiao C, Yang C (2018). Chem Commun.

[46] Yi J, Liang W, Wei X, Yao J, Yan Z, Su D, Zhong Z, Gao G, Wu W, Yang C (2018). Chin Chem Lett.

[47] Alagesan M, Kanagaraj K, Wan S, Sun H, Su D, Zhong Z, Zhou D, Wu W, Gao G, Zhang H (2016). J Photochem Photobiol, A.

[48] Wei X, Yu X, Zhang Y, Liang W, Ji J, Yao J, Rao M, Wu W, Yang C (2019). J Photochem Photobiol, A.

[49] Yang C, Wang Q, Yamauchi M, Yao J, Zhou D, Nishijima M, Fukuhara G, Mori T, Liu Y, Inoue Y (2014). Photochem Photobiol Sci.

[50] Yao J, Yan Z, Ji J, Wu W, Yang C, Nishijima M, Fukuhara G, Mori T, Inoue Y (2014). J Am Chem Soc.

[51] Yang C, Inoue Y (2014). Chem Soc Rev.

[52] Wu J, Liang W, Niu T, Wu W, Zhou D, Fan C, Ji J, Gao G, Men J, Yang Y (2018). Chem Commun.

[53] Leung N L C, Xie N, Yuan W, Liu Y, Wu Q, Peng Q, Miao Q, Lam J W Y, Tang B Z (2014). Chem – Eur J.

[54] Basu B J, Thirumurugan A, Dinesh A R, Anandan C, Rajam K S (2005). Sens Actuators, B.

[55] Zhong F, Zhao J (2017). Dyes Pigm.

[56] Wu X, Zhang Y, Lu Y, Pang S, Yang K, Tian Z, Pei Y, Qu Y, Wang F, Pei Z (2017). J Mater Chem B.

