# Supplementary material for: Synthesis, enantioseparation and photophysical properties of planar-chiral pillar[5]arene derivatives bearing fluorophore fragments
Source: Beilstein J Org Chem. 2019 Jul 18;15:1601–11. doi: 10.3762/bjoc.15.164 (PMC6664395; doi:10.3762/bjoc.15.164)
Supplement: File 1 — Characterization spectra of all compounds, chiral HPLC traces of P5A-Py, CD and UV–vis spectra of the two fractions P5A-Py and the aggregation behaviors of P5A-Py. [file Beilstein_J_Org_Chem-15-1601-s001.pdf]

BEILSTEIN JOURNAL OF ORGANIC CHEMISTRY

Supporting Information

for

Synthesis, enantioseparation and photophysical properties of planar-chiral pillar[5]arene derivatives bearing fluorophore fragments

Guojuan Li, Chunying Fan, Guo Cheng, Wanhua Wu and Cheng Yang

Beilstein J. Org. Chem. **2019,** *15,* 1601–1611. doi:10.3762/bjoc.15.164

Characterization spectra of all compounds, chiral HPLC traces of P5A-Py, CD and UV–vis spectra of the two fractions P5A-Py and the aggregation behaviors of P5A-Py

License and Terms: This is a supporting information file under the terms of the Creative Commons Attribution License (http://creativecommons.org/licenses/by/4.0). Please note that the reuse, redistribution and reproduction in particular requires that the authors and source are credited.
The license is subject to the *Beilstein Journal of Organic Chemistry* terms and conditions: (https://www.beilstein-journals.org/bjoc)

1. Characterization of azide-substitued DPA derivatives and P5A-DPA

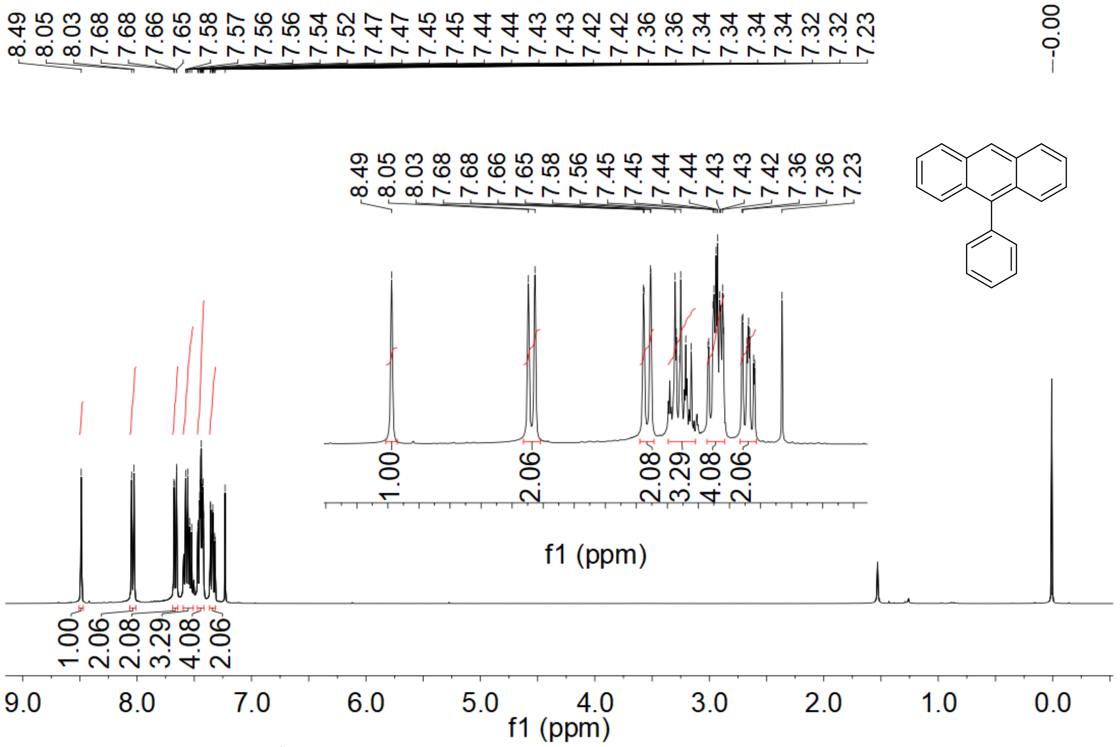

Figure S1: ^{1}H NMR spectrum (400 MHz, chloroform–*d,* room temperature) of **2**.

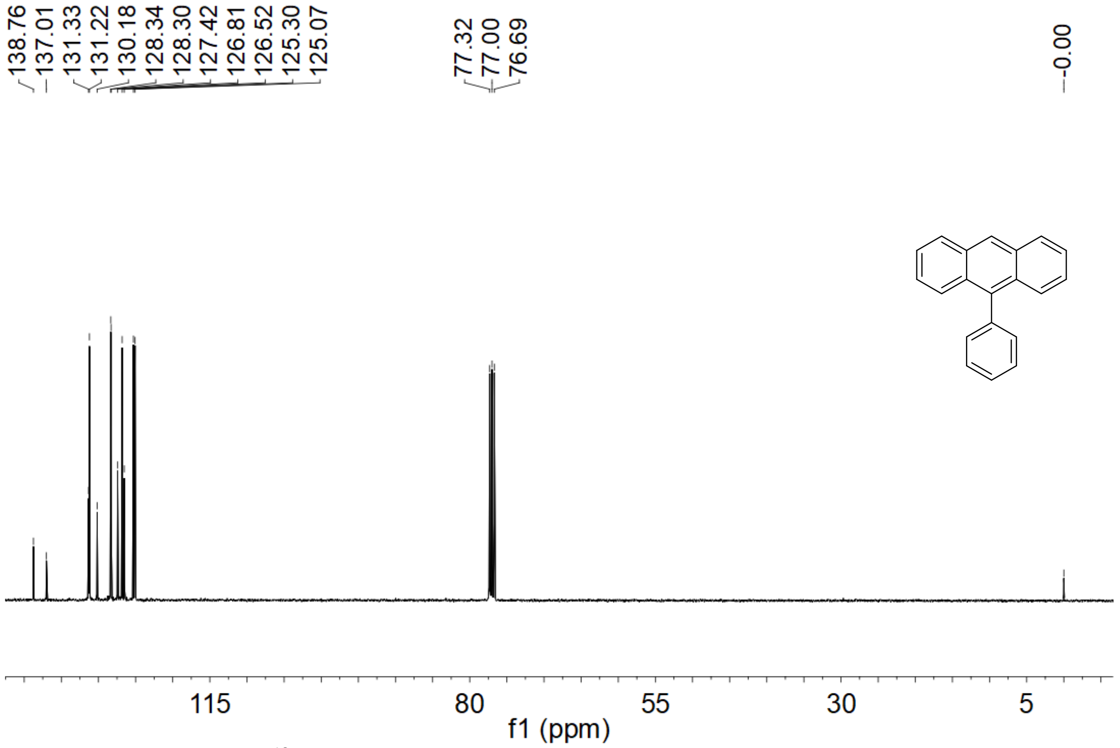

Figure S2: ^{13}C NMR spectrum (100 MHz, chloroform–*d,* room temperature) of **2**.

ESI+

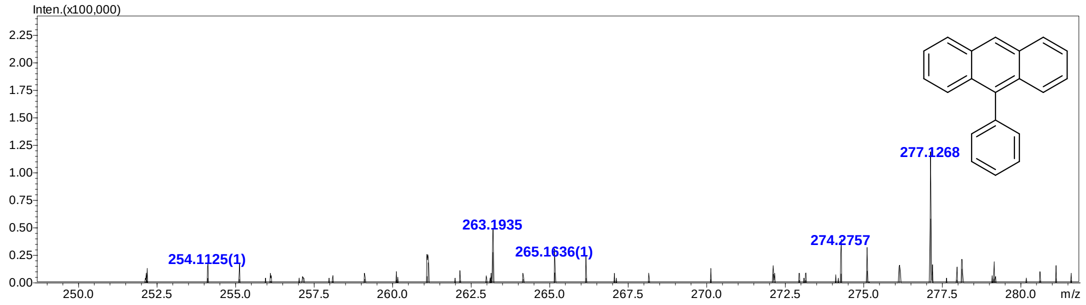

Figure S3: Electrospray ionization mass spectrum of **2**.

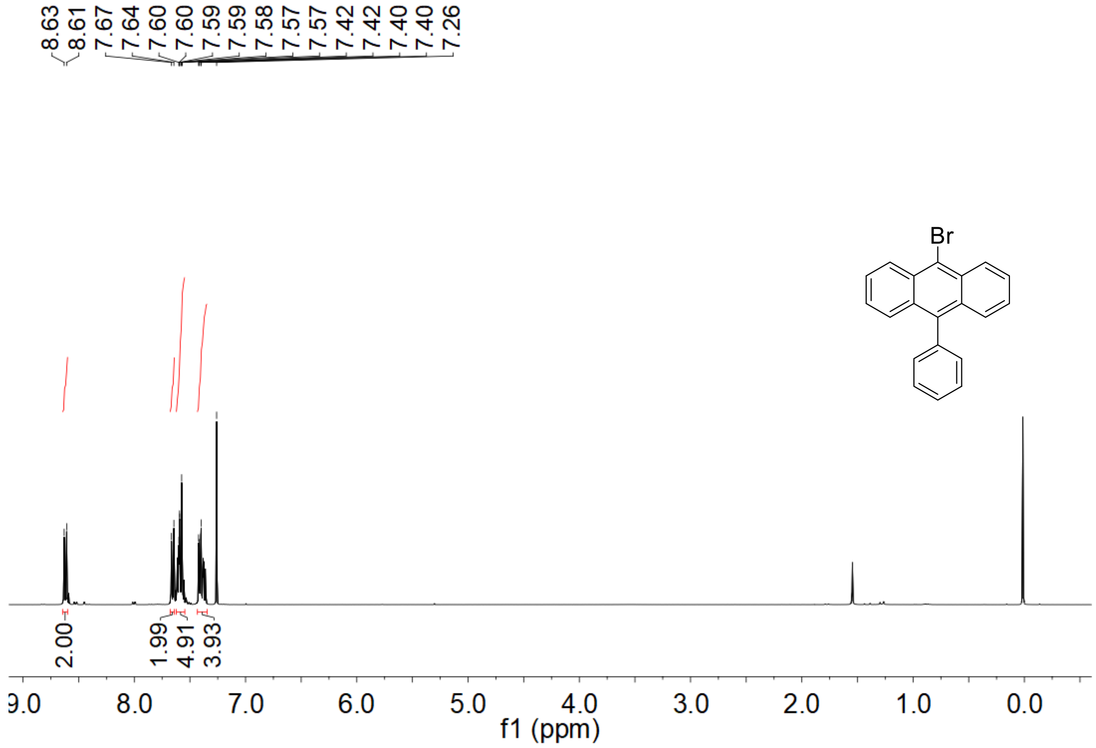

Figure S4: ^{1}H NMR spectrum (400 MHz, chloroform–*d,* room temperature) of **3**.

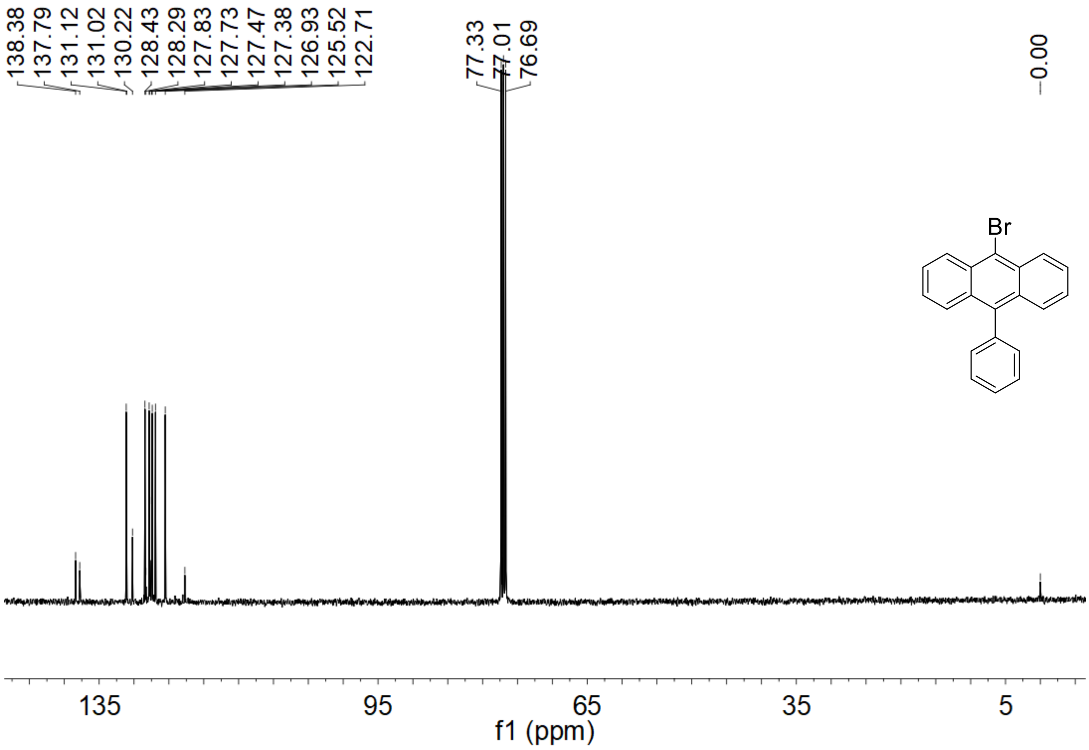

Figure S5: ^{13}C NMR spectrum (100 MHz, chloroform–*d,* room temperature) of **3**.

ESI+

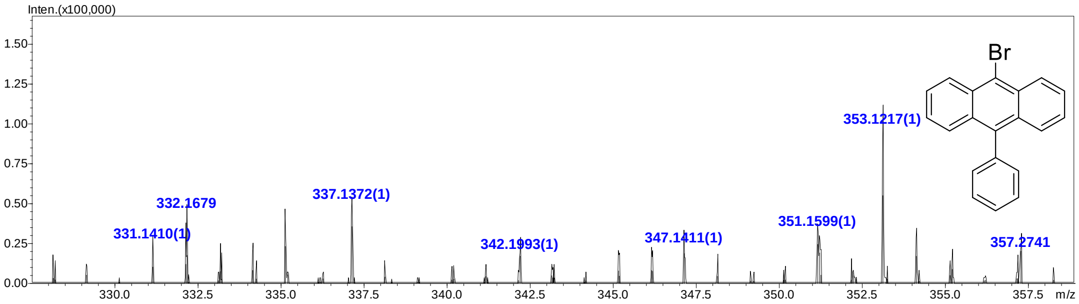

Figure S6: Electrospray ionization mass spectrum of **3**.

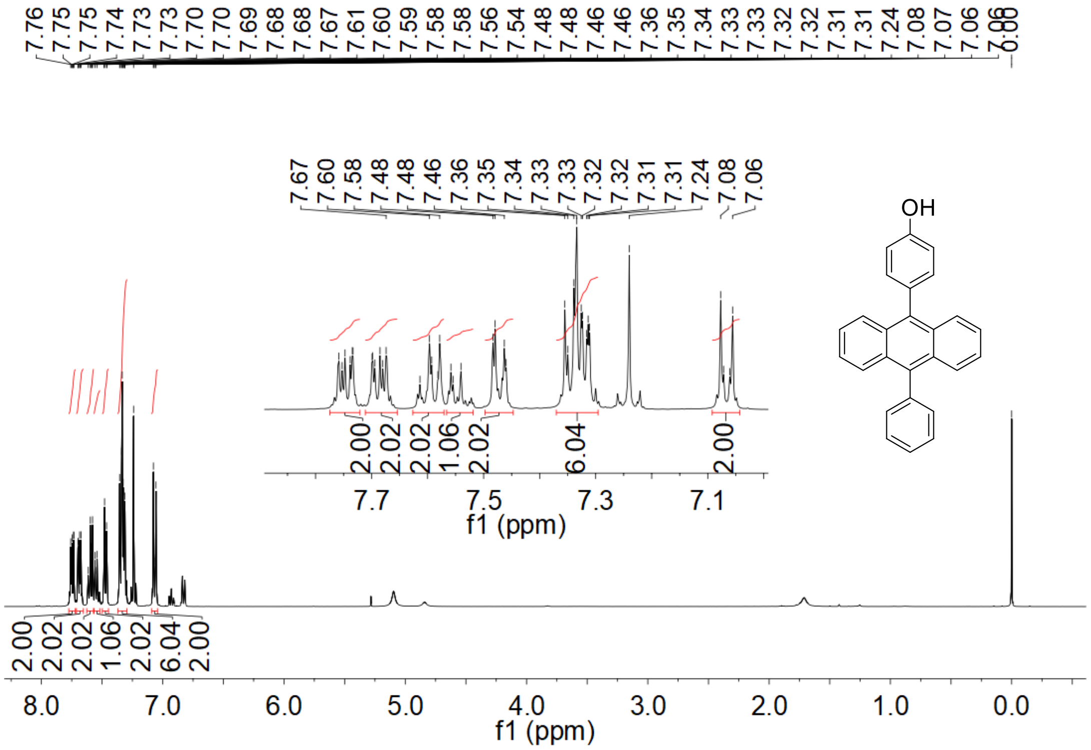

Figure S7: ^{1}H NMR spectrum (400 MHz, chloroform–*d,* room temperature) of **4**.

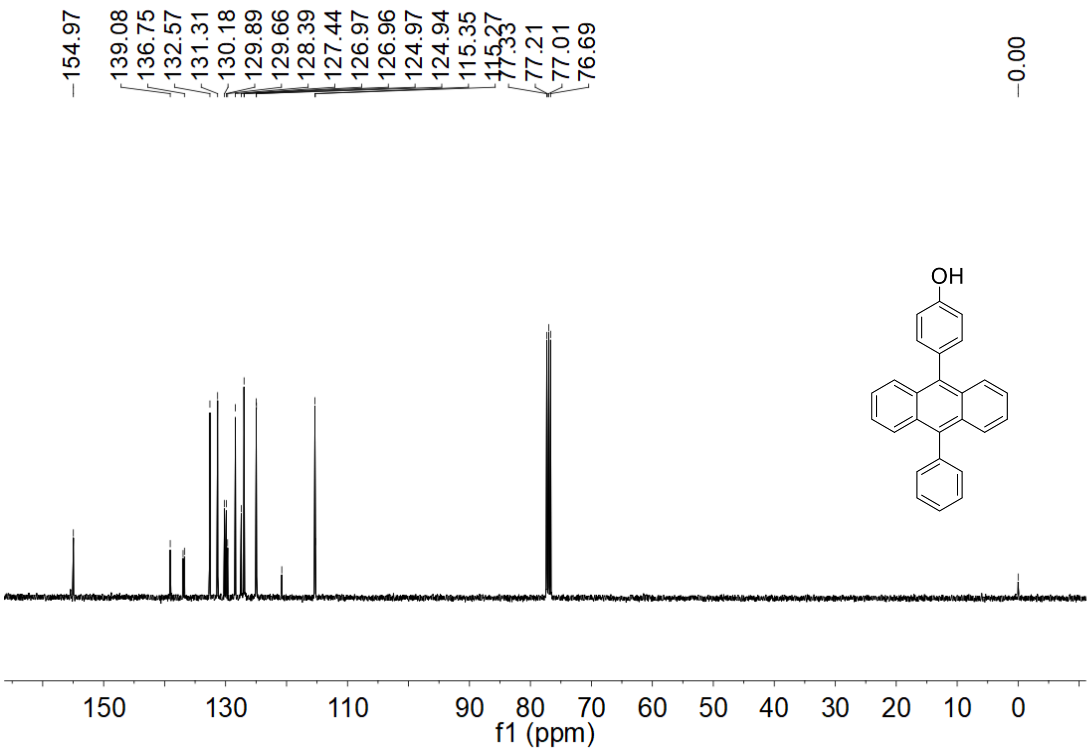

Figure S8: ^{13}C NMR spectrum (100 MHz, chloroform–*d,* room temperature) of **4**.

ESI⁻

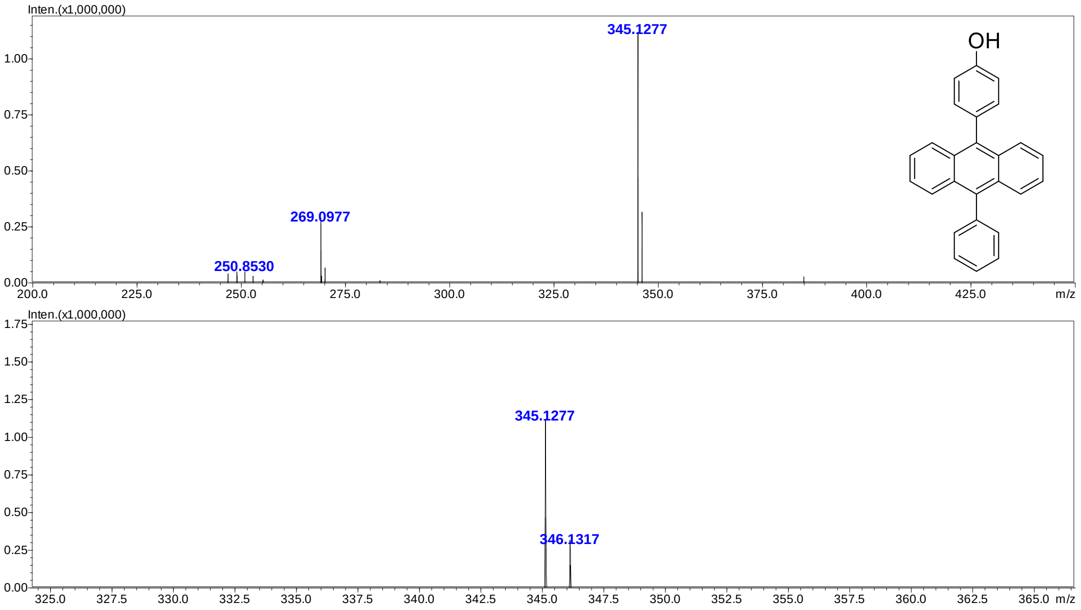

Figure S9: Electrospray ionization mass spectrum of **4**.

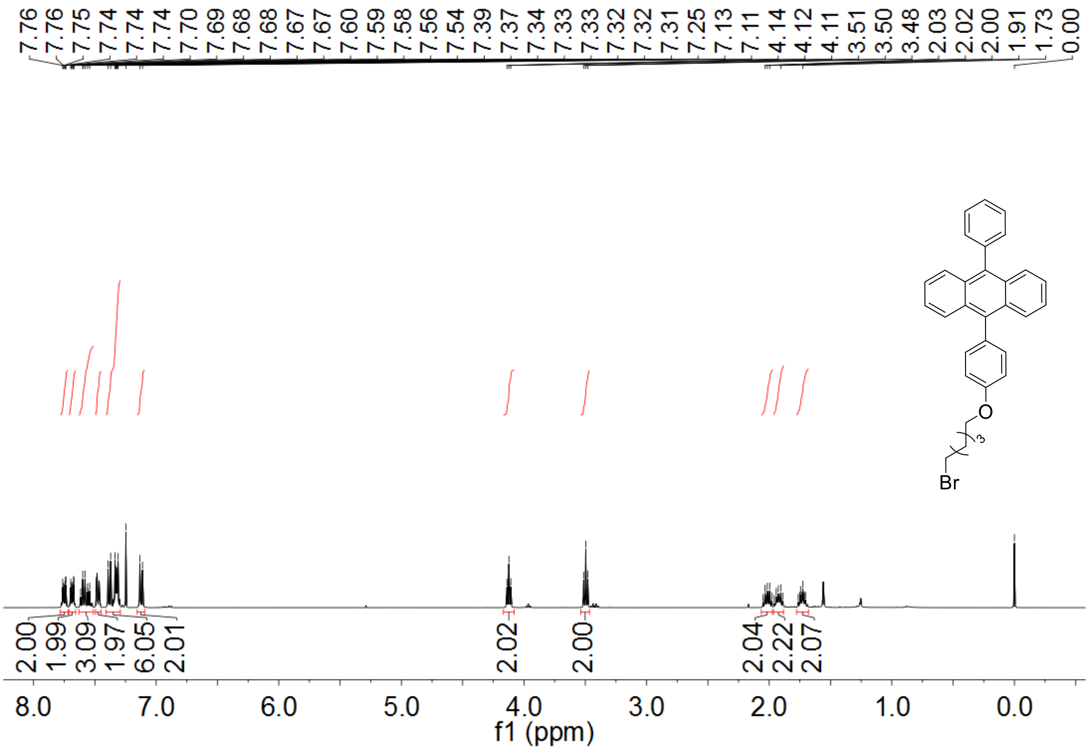

Figure S10: ^{1}H NMR spectrum (400 MHz, chloroform–*d,* room temperature) of **5**.

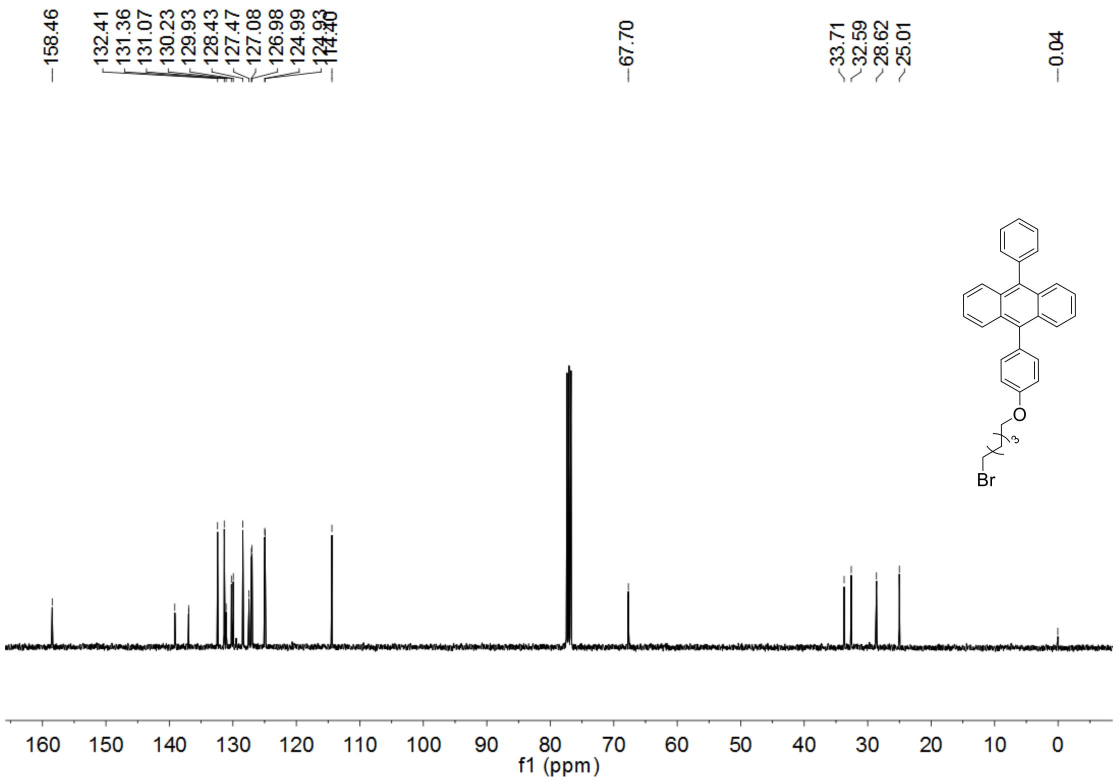

Figure S11: ^{13}C NMR spectrum (100 MHz, chloroform–*d*, room temperature) of **5**.

ESI^{+}

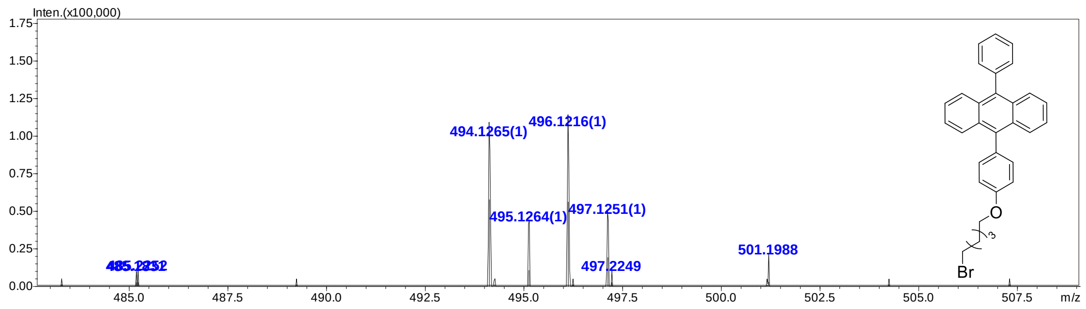

Figure S12: Electrospray ionization mass spectrum of **5**.

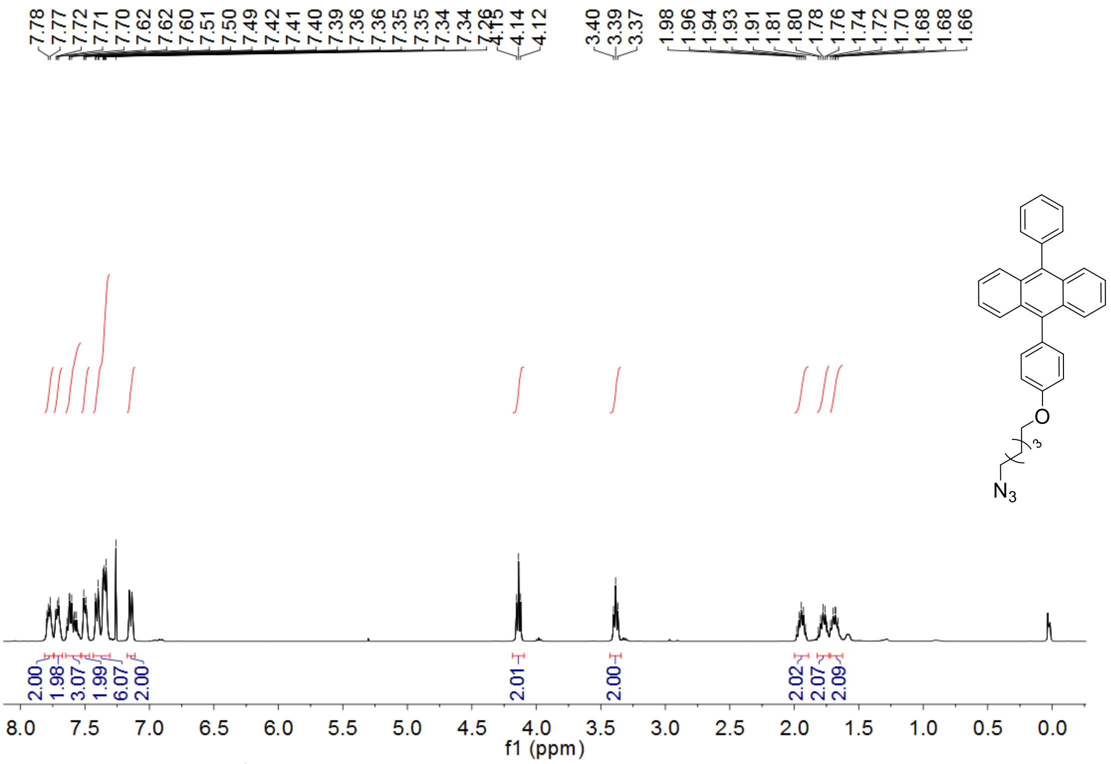

Figure S13: ^{1}H NMR spectrum (400 MHz, chloroform–*d,* room temperature) of **DPA-6**.

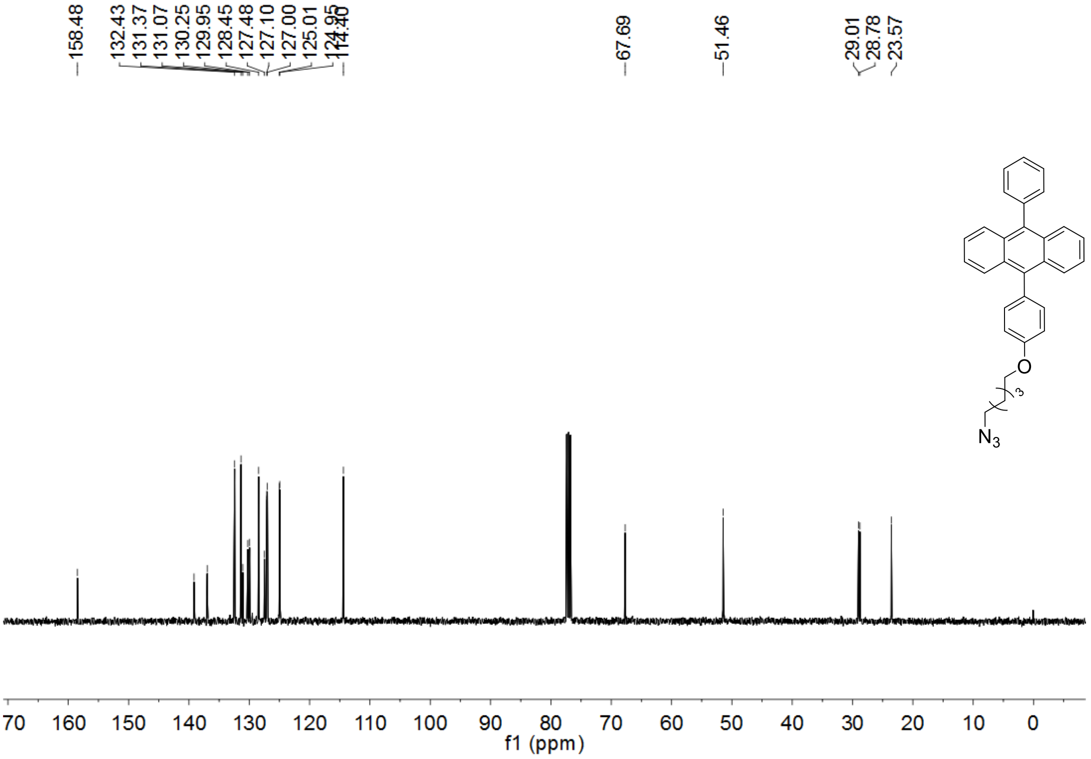

Figure S14: ^{13}C NMR spectrum (100 MHz, chloroform–*d,* room temperature) of **DPA-6**.

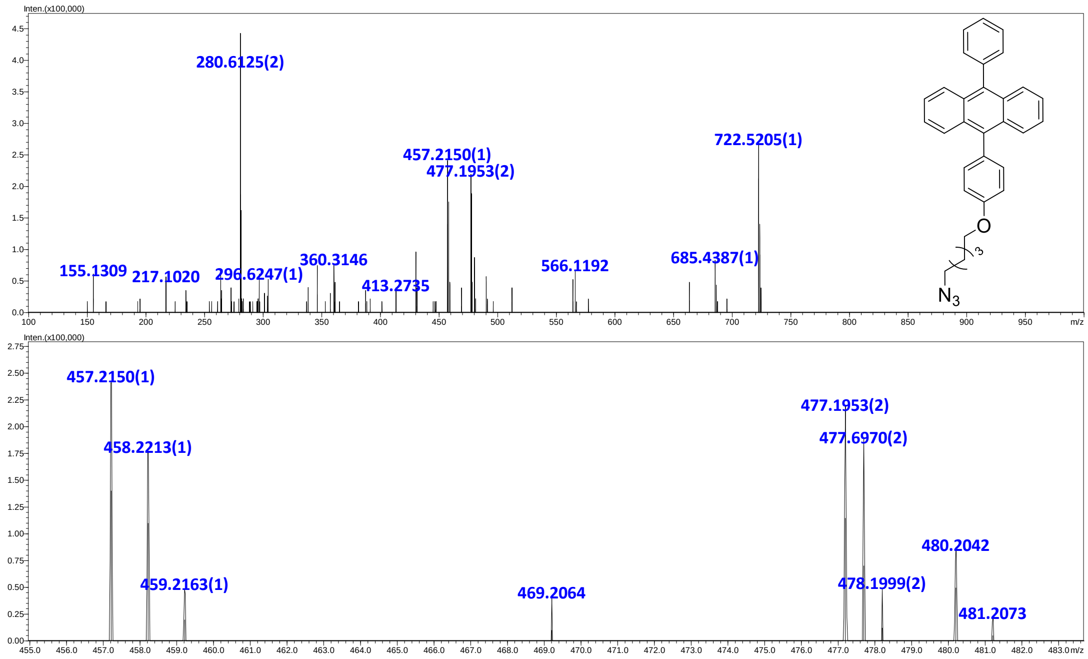

Figure S15: Electrospray ionization mass spectrum of **DPA-6**.

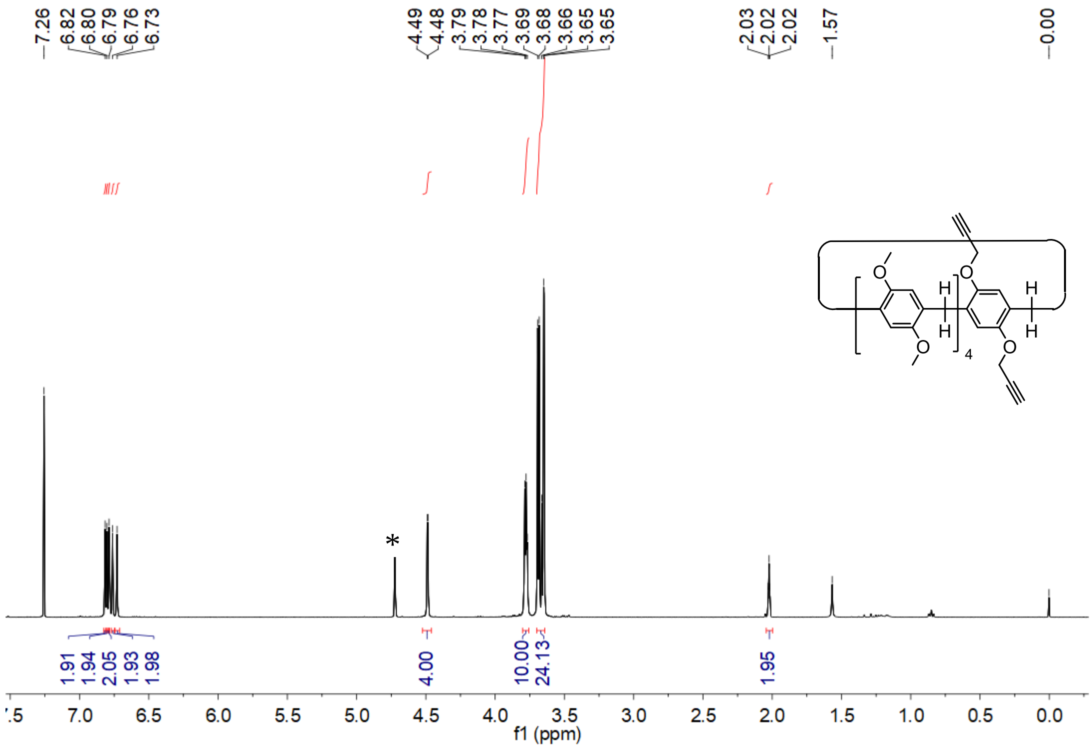

Figure S16: ^{1}H NMR spectrum (400 MHz, chloroform–*d,* room temperature) of **P5A**, *is the signal of DCM which was included in the cavity of the host.

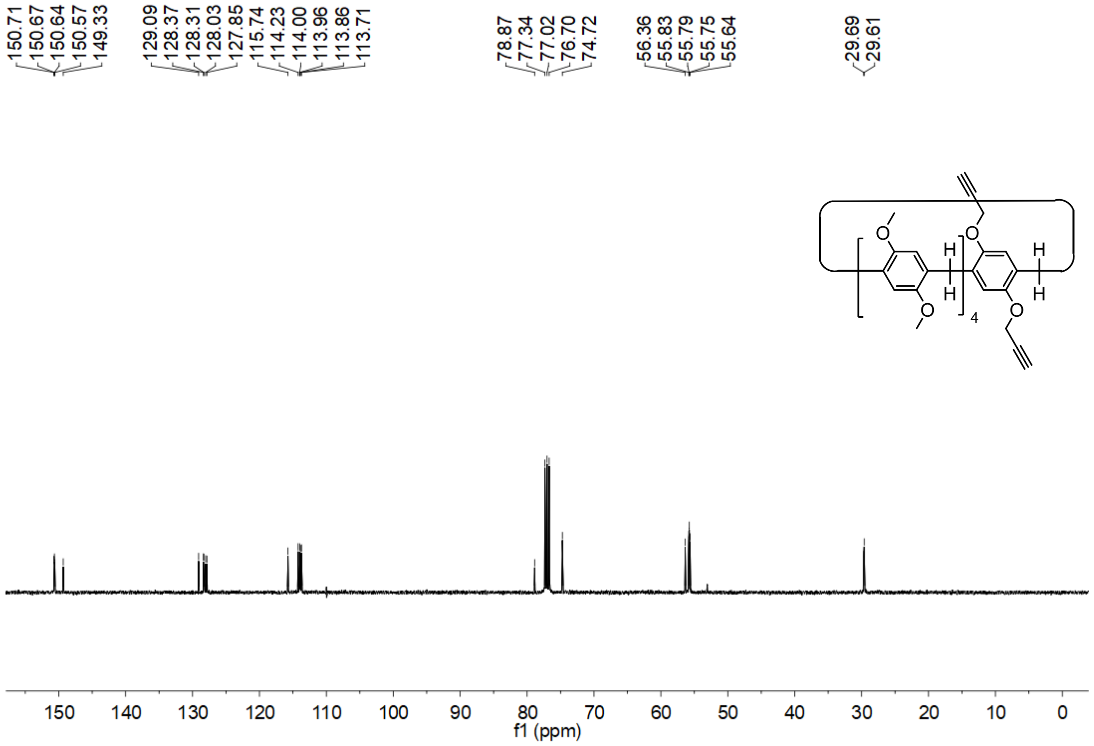

Figure S17:^{13}C NMR spectrum (100 MHz, chloroform–*d,* room temperature) of **P5A**.

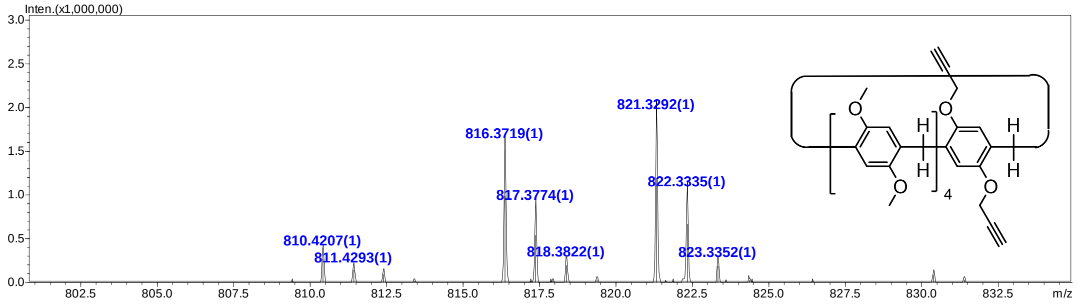

Figure S18: Electrospray ionization mass spectrum of **P5A**.

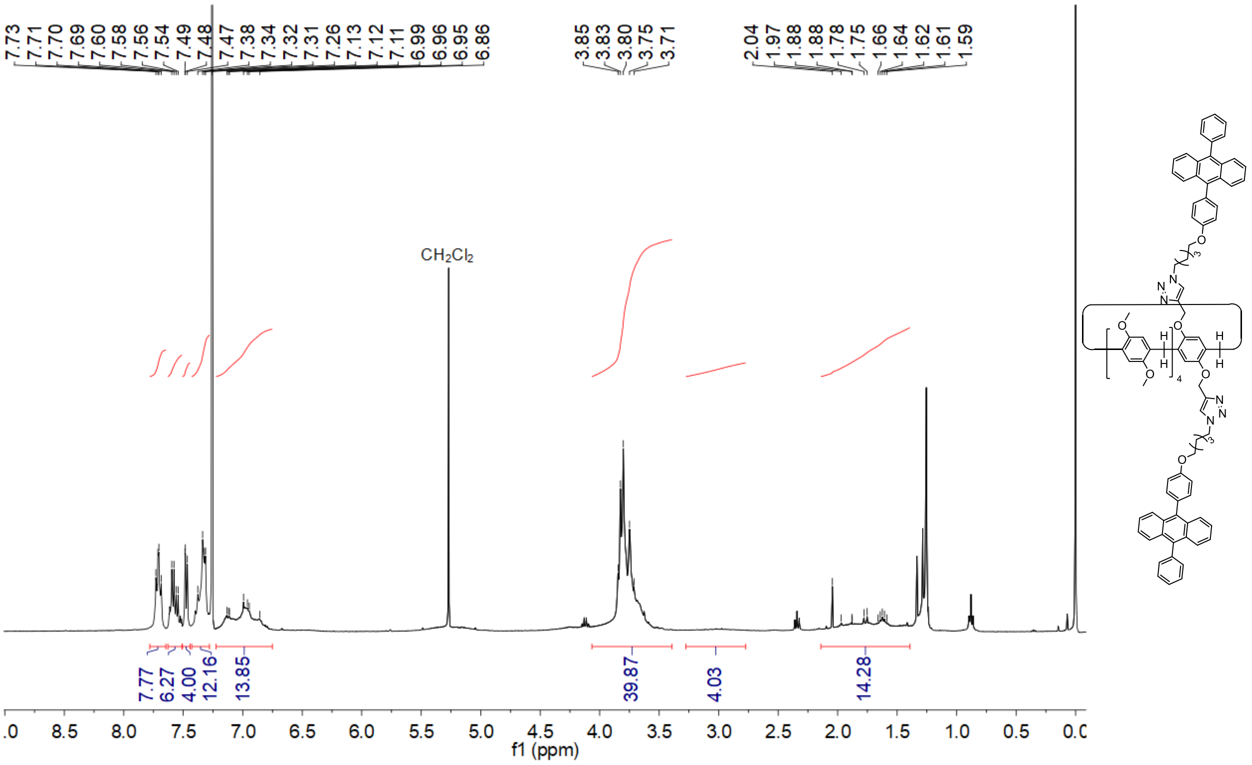

Figure S19: ^{1}H NMR spectrum (600 MHz, chloroform–*d,* room temperature) of **P5A-DPA**.

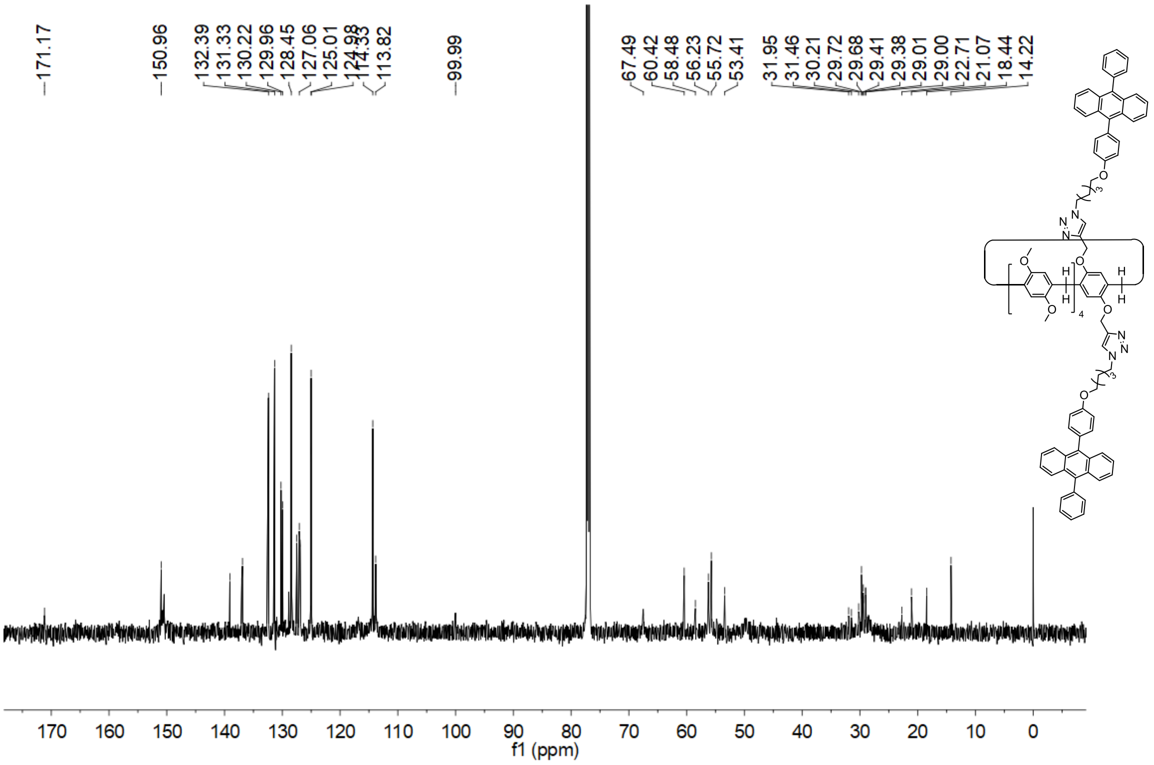

Figure S20: ^{13}C NMR spectrum (151 MHz, chloroform–*d,* room temperature) of **P5A-DPA**.

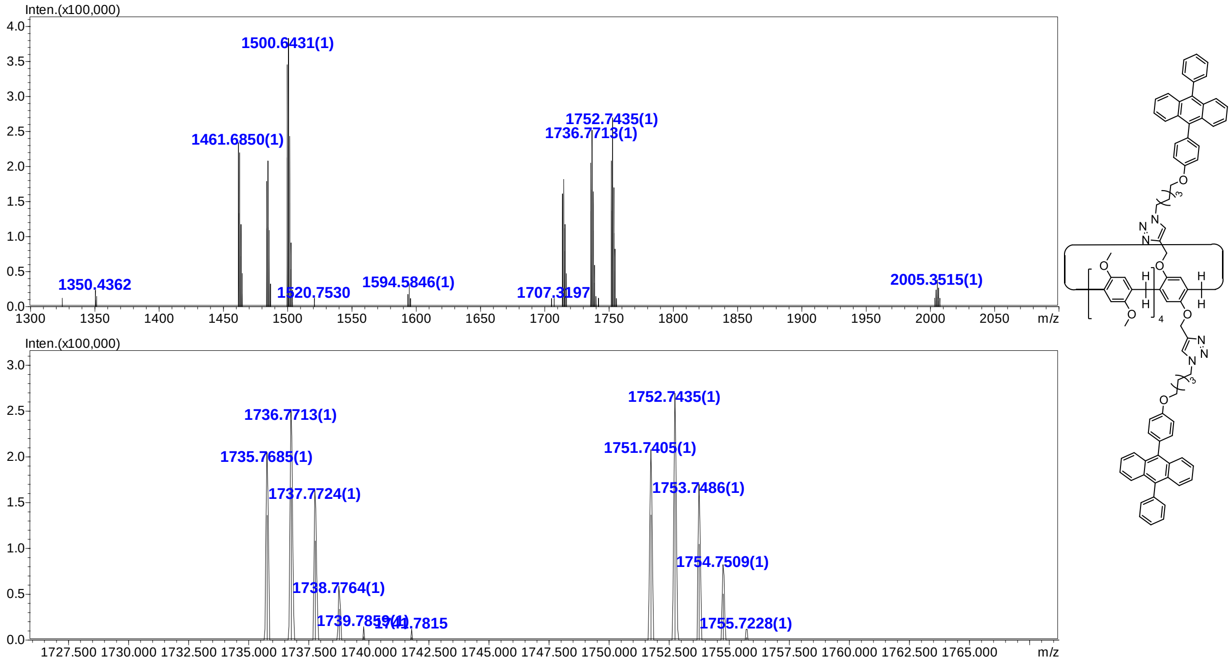

Figure S21: Electrospray ionization mass spectrum of **P5A-DPA**.

2. Conformational characteristics of P5A-Py

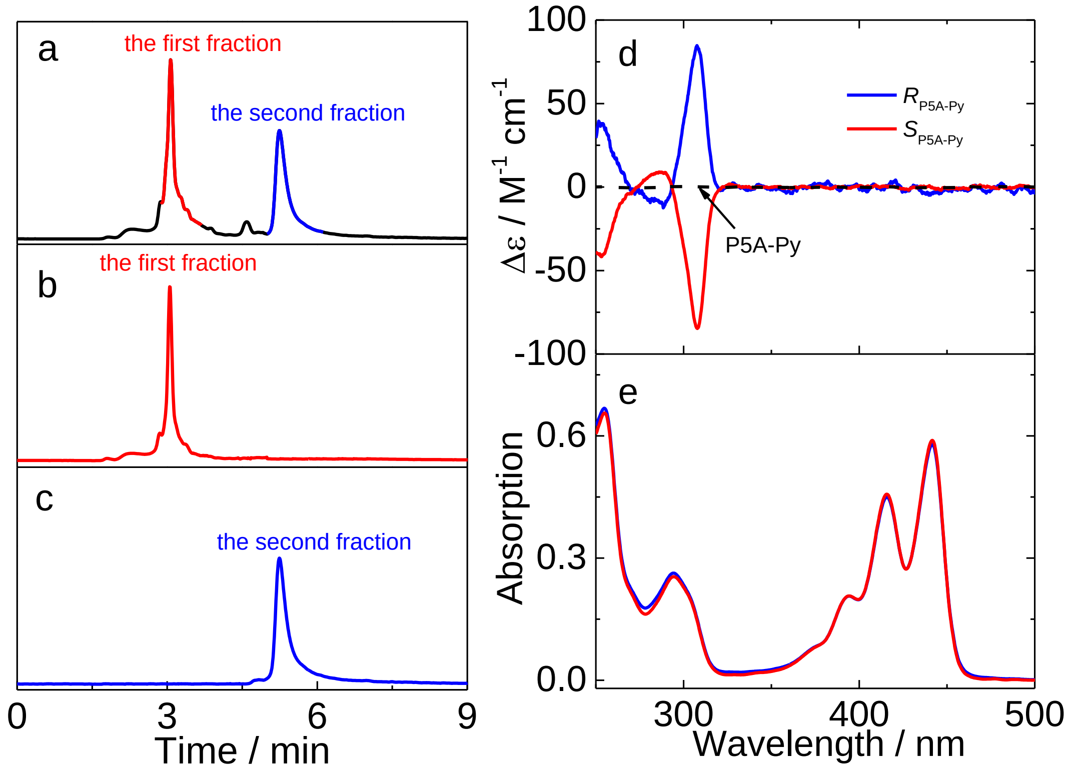

Figure S22: (a) Chiral HPLC traces of **P5A-Py**, (b) (c) the first and second fractions of **P5A-Py**, detected by UV at 295 nm. (Conditions; column: DAICEL CHIRALPAK IA; mobile phase: hexane/dichloromethane 35:65; flow rate = 4.0 mL/min; temperature: 25 °C); (d) CD and (e) UV–vis spectra of the first and second fractions (10 μM) in $CHCl_3$ at 25 °C.

3. Aggregation behaviors of P5A-Py

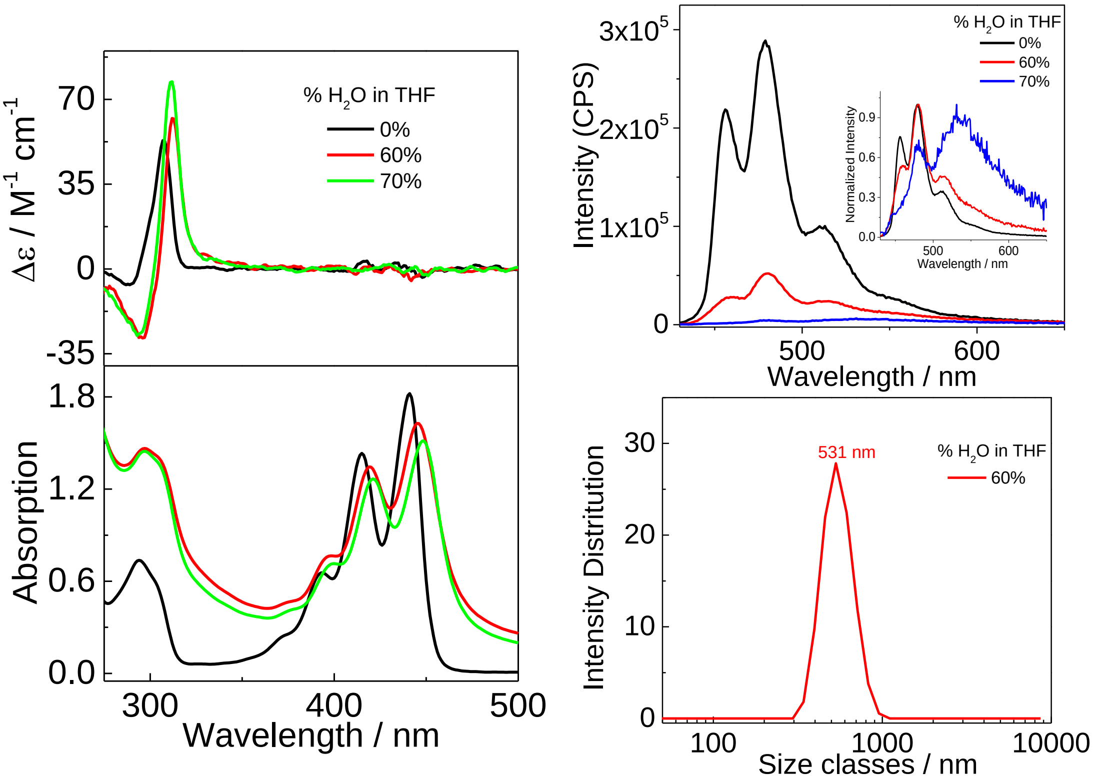

Figure S23: (a) CD, (b) UV–vis and (c) fluorescence spectra of the $R_{\textbf{P5A-Py}}$ (20 μM) in pure THF, 60% and 70% water in THF; inset: the normalized fluorescence spectrum. (d) DLS measurements of the aggregated $R_{\textbf{P5A-Py}}$ obtained from the mixture of THF/H_2O (60%).
